# The histone H3/H4 chaperone CHAF1B prevents the mislocalization of CENP-A for chromosomal stability

**DOI:** 10.1242/jcs.260944

**Published:** 2023-05-31

**Authors:** Roshan L. Shrestha, Vinutha Balachandra, Jee Hun Kim, Austin Rossi, Pranathi Vadlamani, Subhash Chandra Sethi, Laurent Ozbun, Shinjen Lin, Ken Chin-Chien Cheng, Raj Chari, Tatiana S. Karpova, Gianluca Pegoraro, Daniel R. Foltz, Natasha J. Caplen, Munira A. Basrai

**Affiliations:** ^1^ Yeast Genome Stability Section, Genetics Branch, Center for Cancer Research (CCR), National Cancer Institute (NCI), National Institutes of Health (NIH), Bethesda, MD 20892, USA; ^2^ Department of Biochemistry and Molecular Genetics, Northwestern University, Chicago, IL 60611, USA; ^3^ High-Throughput Imaging Facility (HiTIF), Laboratory of Receptor Biology and Gene Expression, CCR, NCI, NIH, Bethesda, MD 20892, USA; ^4^ Functional Genomics Facility, National Center for Advancing Translational Sciences, NIH, Bethesda, MD 20892, USA; ^5^ Genome Modification Core, Laboratory Animal Sciences Program, Frederick National Laboratory for Cancer Research, Frederick, MD 21701, USA; ^6^ Optical Microscopy Core, Laboratory of Receptor Biology and Gene Expression, CCR, NCI, NIH, Bethesda, MD 20892, USA; ^7^ Functional Genetics Section, Genetics Branch, CCR, NCI, NIH, Bethesda, MD 20892, USA

**Keywords:** CENP-A, Chromosomal instability, CHAF1B, DAXX

## Abstract

Restricting the localization of the evolutionarily conserved centromeric histone H3 variant CENP-A to centromeres prevents chromosomal instability (CIN). The mislocalization of CENP-A to non-centromeric regions contributes to CIN in yeasts, flies and human cells. Even though overexpression and mislocalization of CENP-A have been reported in cancers, the mechanisms responsible for its mislocalization remain poorly understood. Here, we used an imaging-based high-throughput RNAi screen to identify factors that prevent mislocalization of overexpressed YFP-tagged CENP-A (YFP–CENP-A) in HeLa cells. Among the top five candidates in the screen – the depletion of which showed increased nuclear YFP–CENP-A fluorescence – were the histone chaperones CHAF1B (or p60) and CHAF1A (or p150). Follow-up validation and characterization experiments showed that CHAF1B-depleted cells exhibited CENP-A mislocalization, CIN phenotypes and increased enrichment of CENP-A in chromatin fractions. The depletion of DAXX, a histone H3.3 chaperone, suppressed CENP-A mislocalization and CIN in CHAF1B-depleted cells. We propose that in CHAF1B-depleted cells, DAXX promotes mislocalization of the overexpressed CENP-A to non-centromeric regions, resulting in CIN. In summary, we identified regulators of CENP-A localization and defined a role for CHAF1B in preventing DAXX-dependent CENP-A mislocalization and CIN.

## Teaser

**Summary:** The histone H3/H4 chaperone CHAF1B prevents mislocalization of CENP-A to non-centromeric regions, thus preventing chromosomal instability.

## INTRODUCTION

Aneuploidy is a hallmark of many cancers and a significant driver of tumorigenesis. Chromosomal instability (CIN), characterized by an unequal distribution of chromosomes into two daughter cells and/or structural rearrangements of the genome, initiates aneuploidy (Thompson and Compton, 2008). One of the key chromatin structures necessary to maintain chromosomal stability is the centromere, which serves as a site for assembly of the kinetochore, which in turn mediates kinetochore–microtubule attachments and spindle assembly checkpoint functions (Cleveland et al., 2003). The evolutionarily conserved histone H3 variant CENP-A (Cse4 in budding yeast, Cnp1 in fission yeast, CID in fruit fly) is essential for kinetochore assembly and chromosomal stability. CENP-A serves as an epigenetic marker to distinguish centromeric from non-centromeric nucleosomes and is distributed to the sister chromatids during DNA replication in S phase (Zasadzinska et al., 2018). Recruitment of CENP-A at the centromeres in the G1 phase of the cell cycle is mediated by the CENP-A-specific chaperone, holiday junction recognition protein (HJURP), and its interaction with Mis18β or condensin II (Barnhart-Dailey et al., 2017; Nardi et al., 2016). Moreover, epigenetic mechanisms and post-translational modifications such as phosphorylation, monoubiquitination, acetylation and trimethylation of CENP-A also regulate its deposition at centromeric chromatin (McKinley and Cheeseman, 2016; Niikura et al., 2019; Sathyan et al., 2017; Silva et al., 2012; Stankovic et al., 2017).

CENP-A overexpression and mislocalization have been observed in many cancers, correlating with disease stage, increased risk of disease progression and poor patient survival (Saha et al., 2020; Smith and Sheltzer, 2022; Wang et al., 2021; Xu et al., 2020; Zhang et al., 2016). We previously provided the first evidence showing that mislocalization of Cse4 contributes to CIN in budding yeast, and comparable results have been reported in fission yeast and fly (Au et al., 2008; Choi et al., 2012; Heun et al., 2006). Studies focused on the causes of Cse4 mislocalization have defined a role for the histone chaperone complex HIR (HIRA in humans) and post-translational modifications of Cse4, such as ubiquitination, sumoylation and phosphorylation, which regulate Cse4 levels by proteolysis, and which prevent its mislocalization to non-centromeric regions and subsequent CIN in budding yeast (Hewawasam et al., 2010; Ranjitkar et al., 2010; Au et al., 2013; Ciftci-Yilmaz et al., 2018; Deyter and Biggins, 2014; Eisenstatt et al., 2020; Ohkuni et al., 2018, 2020, 2022).

Similar to results obtained in budding yeast, we have shown that the mislocalization of overexpressed CENP-A contributes to CIN in aneuploid (HeLa), diploid (RPE1) and pseudodiploid (DLD1) human cell lines (Shrestha et al., 2017, 2021). Comprehensive analysis of mitotic phenotypes showed a concentration-dependent effect of CENP-A overexpression on chromosome segregation defects and a higher incidence of micronuclei: both features of CIN. The CIN phenotypes were due to defects in kinetochore integrity as CENP-A-overexpressing cells showed reduced levels of kinetochore proteins at centromeric chromatin and unstable kinetochore–microtubule attachments (Shrestha et al., 2021). Furthermore, our results showed that CENP-A overexpression contributes to aneuploidy with karyotypic heterogeneity in DLD1 cells and xenograft tumor models (Shrestha et al., 2021). Finally, we and others have shown that the histone H3.3 chaperone DAXX contributes to the mislocalization of overexpressed CENP-A in HeLa cells (Lacoste et al., 2014; Shrestha et al., 2017).

Despite the clinical significance of CENP-A overexpression and mislocalization, the mechanisms that prevent CENP-A mislocalization to non-centromeric regions in human cells are not fully characterized. In this study, we performed an imaging-based high-throughput RNAi screen to identify proteins that regulate the expression and nuclear localization of CENP-A, thus maintaining chromosomal stability. As we have previously shown that increased nuclear levels of CENP-A correlate with its mislocalization (Shrestha et al., 2017), increased nuclear YFP–CENP-A fluorescence intensity provided a proxy measurement for CENP-A mislocalization in this assay. Nuclear YFP–CENP-A fluorescence was used as a reporter to screen a library of 521 chromatin factors, which led to the identification of histone chaperones [CHAF1B (or p60), CHAF1A (or p150) and HIRA], multiple components of the NuA4 histone acetyltransferase complex [EP400, KAT5 (or TIP60) and TRRAP] and a component of the Skp1, Cullin and F-box (SCF) ubiquitin ligase (SKP1) among the top candidates in the screen.

CHAF1A, CHAF1B and RbAp48 (or RBBP4) in a 1:1:1 stoichiometry form the CAF-1 complex. As CHAF1A and CHAF1B were among the top five candidate genes in the screen, we decided to pursue in-depth characterization of the potential role of CHAF1B in preventing the mislocalization of CENP-A. CHAF1B interacts with ASF1a, an H3/H4 chaperone that binds H3/H4 heterodimers directly (English et al., 2006). During S phase, the CHAF1B–ASF1a–H3/H4 subcomplex binds first with CHAF1A, which in turn interacts with the DNA replication clamp loader PCNA (Shibahara and Stillman, 1999). Hence, the CAF-1 complex facilitates the delivery of newly synthesized H3/H4 dimers to the replication fork during DNA synthesis. In addition to its role in S phase, CHAF1B also regulates nucleotide excision repair synthesis following DNA damage and recruitment of ubiquitinated H2A to DNA damage foci (De Koning et al., 2007; Zhu et al., 2009). Here, we define a previously unreported role for CHAF1B in preventing CENP-A mislocalization and CIN. Our results further show that co-depletion of CHAF1B and DAXX suppresses CENP-A mislocalization and CIN. Our studies provide insights into the pathways that prevent CENP-A mislocalization and CIN and advance our understanding for how defects in these pathways might contribute to aneuploidy in CENP-A-overexpressing cancers.

## RESULTS

### Development of an imaging-based high-throughput screen to identify genes that regulate nuclear localization of CENP-A

We have previously described the generation of HeLa cells overexpressing either low or high levels of YFP-tagged CENP-A: HeLa^YFP–CENP-A-low^ and HeLa^YFP–CENP-A-high^, respectively (Shrestha et al., 2017). Using these reporter cell lines, we showed that the nuclear intensity of CENP-A correlates with its mislocalization to non-centromeric regions (Shrestha et al., 2017). Based on these results, we hypothesized that nuclear YFP–CENP-A fluorescence intensity can function as a proxy measurement for CENP-A mislocalization and as a readout for an imaging-based functional RNAi screen focused on identifying factors that prevent the mislocalization of CENP-A to non-centromeric regions.

**Fig. 1.**
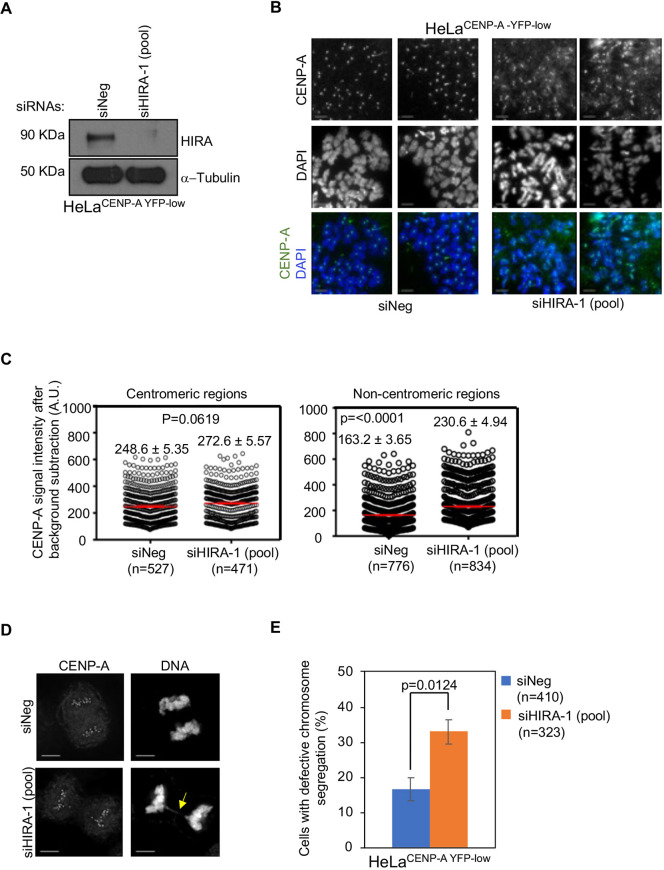
**Depletion of HIRA, a positive control for the imaging-based screen, shows the mislocalization of CENP-A and CIN phenotypes in HeLa^YFP–CENP-A-low^ cells.** (A) Western blots of lysates prepared from HeLa^YFP–CENP-A-low^ cells transfected with the indicated siRNAs for 72 h and analyzed using the antibodies as indicated. (B) Representative images of metaphase chromosome spreads showing the localization of CENP-A at centromeric and non-centromeric regions in HeLa^YFP–CENP-A-low^ cells transfected with the indicated siRNAs. Metaphase chromosome spreads were prepared 72 h post transfection, and cells were immunostained using an antibody against CENP-A and stained with DAPI. Scale bars: 5 µm. (C) Quantification of CENP-A signal intensities at centromeric (left) and non-centromeric (right) regions in metaphase chromosome spreads of HeLa^YFP–CENP-A-low^ cells transfected with the indicated siRNAs. Each circle represents a spot on a centromeric or non-centromeric region. *n* denotes the number of chromosomes analyzed. Red lines indicated mean±s.e.m. for YFP signal intensities across areas measured in the number of spots indicated as *n* from three independent experiments. A.U., arbitrary units. (D,E) Immunostained images (D) and bar chart (E) showing the proportion of cells with defective chromosome segregation in HeLa^YFP–CENP-A-low^ cells transfected with the indicated siRNAs. The yellow arrow shows lagging chromosomes. Error bars represent s.e.m. across three independent experiments. *n* denotes the number of cells analyzed from three experiments. *P*-values shown in C, E were calculated using unpaired, two-tailed *t*-test.

To develop our assay system, we first validated an appropriate positive-control gene target. We have shown previously that the H3.3 chaperone HIRA prevents CENP-A mislocalization in budding yeast, and similar observations have been reported in the colorectal cancer cell line SW480 (Ciftci-Yilmaz et al., 2018; Nye et al., 2018). We therefore assessed whether siRNA-mediated depletion of HIRA leads to the mislocalization of CENP-A in HeLa^YFP–CENP-A-low^ cells. Using western blotting, we confirmed an efficient depletion of HIRA in HeLa^YFP–CENP-A-low^ cells transfected with an siRNA targeting *HIRA* [siHIRA-1(pool)] (Fig. 1A). We next assessed the effects of HIRA depletion on the localization on CENP-A on mitotic chromosomes. Metaphase chromosome spreads of HeLa^YFP–CENP-A-low^ cells immunostained using an anti-CENP-A antibody showed the mislocalization of CENP-A to non-centromeric regions following depletion of HIRA (Fig. 1B,C). Previously, we have also reported that the mislocalization of overexpressed CENP-A contributes to CIN phenotypes, such as chromosome segregation defects in HeLa and DLD1 cells (Shrestha et al., 2017, 2021). Hence, we examined whether siRNA-mediated depletion of HIRA, which leads to the mislocalization of CENP-A, also contributes to CIN in HIRA-depleted HeLa^YFP–CENP-A-low^ cells. Indeed, the results of these experiments showed that HIRA-depleted HeLa^YFP–CENP-A-low^ cells exhibited a significantly higher proportion of cells with defective chromosome segregation compared to that of control cells (Fig. 1D,E). Collectively, these results support the use of siRNA reagents targeting *HIRA* as an appropriate positive control to assay increased YFP–CENP-A nuclear fluorescence intensity.

**Fig. 2.**
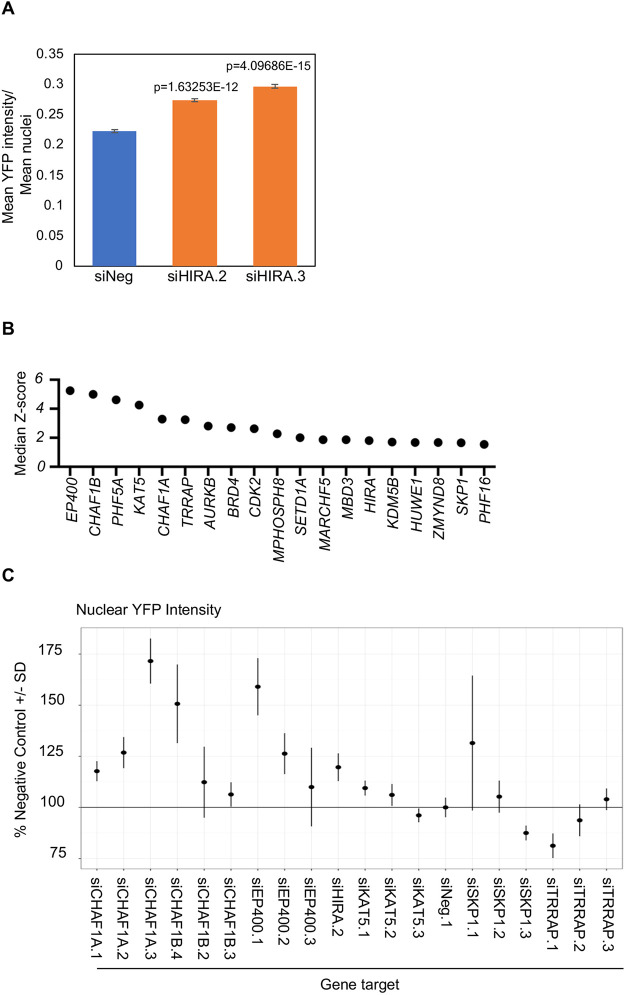
**High-throughput RNAi screen identifies regulators of CENP-A mislocalization.** (A) Bar chart showing the ratio between mean YFP signal intensities and mean number of nuclei in HeLa^YFP–CENP-A-high^ cells transfected with the indicated siRNAs for 72 h in 384-well plates. (B) Median Z-score of lead candidate genes obtained from the primary screen of an siRNA chromatin library for increased nuclear YFP intensity. (C) Secondary analysis of lead candidate siRNAs selected from the primary RNAi screen of genes that regulate epigenetic processes. The nuclear YFP intensity data are presented relative to that of cells transfected with a scrambled siRNA (siNeg.1). The results from siHIRA.2 are shown as a positive control.

To perform high-throughput siRNA screening in a multi-well format (i.e. in 384-well plates) using YFP signal intensity as the readout, we used HeLa^YFP–CENP-A-high^ cells (Shrestha et al., 2017), so that the YFP signals could be captured without requiring immunostaining using an anti-CENP-A antibody. We assessed the YFP–CENP-A nuclear intensity in HeLa^YFP–CENP-A-high^ cells transfected with either of two independent siRNAs targeting different sequences of *HIRA* (siHIRA.2 and siHIRA.3) (Table S1) in a high-throughput format. In brief, we captured images of fixed and DAPI-stained HeLa^YFP–CENP-A-high^ cells 72 h post siRNA transfection using a high-throughput spinning-disk microscope (see Materials and Methods for further details). We used a high-content image-analysis pipeline to measure the mean nuclear fluorescent intensity of YFP signal in nucleus masks generated by nuclear segmentation based on the DAPI channel. The measurement of the ratio between the mean YFP signal intensity to the mean number of nuclei confirmed that HeLa^YFP–CENP-A-high^ cells transfected with *HIRA* siRNAs exhibited significantly higher CENP-A signal intensities compared to those observed in control cells (Fig. 2A). We thus used siHIRA.2 and siHIRA.3 as positive-control siRNAs for our imaging-based screen.

### An imaging-based high-throughput screen identifies multiple members of the histone chaperone complex as putative regulators of CENP-A nuclear localization

Using HeLa^YFP–CENP-A-high^ cells, we screened an siRNA library targeting 521 human genes (Table S2) encoding, among other protein families, histone methyltransferases, demethylases, acetylases, deacetylases, histone chaperones, chromatin remodelers and E3 ubiquitin ligases. The library included three different siRNA oligonucleotides per gene, with one siRNA per well. We performed two independent biological replicates of the screen with relevant quality-control metrics (Fig. 2A). Following image processing, quantification of fluorescence intensities and statistical analyses (see Materials and Methods), we first calculated Z-scores for individual siRNA oligonucleotides and then used gene-level median Z-scores (i.e. the Z-score values of the second most potent siRNA oligonucleotide out of three) to rank the results of the screen (Table S2). RNAi silencing of genes encoding multiple components of the NuA4 histone acetyltransferase complex (EP400, KAT5 and TRRAP), histone chaperones (CHAF1B, CHAF1A and, as expected, HIRA) and SKP1, a component of the SCF ubiquitin ligase, led to the highest increase in YFP nuclear intensity in our assay (Fig. 2B; Table S2).

To confirm the results of the primary screen, we used the same imaging-based assay in a secondary validation experiment employing siRNAs oligonucleotides synthesized using a different chemistry and targeting different sequences corresponding to the candidate genes of interest, specifically, *EP400*, *KAT5*, *TRRAP*, *CHAF1A, CHAF1B*, and *SKP1* (three siRNAs oligonucleotides per gene) (Table S1). We used siHIRA.2 as a positive control (Fig. 2A) and the HeLa^YFP–CENP-A-high^ cells for secondary validation. The results of these validation experiments demonstrated higher nuclear levels of YFP–CENP-A after transfection of three independent siRNA oligonucleotides per gene for five of the six genes (Fig. 2C). Reassuringly, the identification of SKP1 as a putative regulator of CENP-A localization is consistent with our previous results in budding yeast that demonstrated a role for the F-box proteins Met30 and Cdc4, subunits of the SCF complex, in preventing mislocalization of Cse4 (Au et al., 2020). Moreover, the identification of multiple members of the same protein complex, specifically the NuA4 and the CAF-1 complexes, and with four different siRNA oligonucleotides synthesized with a different chemistry, represents a novel finding and is highly unlikely owing to RNAi off-target effects, indicating a potential role for these complexes as physiological regulators of CENP-A localization.

### CHAF1B-depleted cells show the mislocalization of CENP-A to non-centromeric regions and CIN phenotypes

The identification of two components of the replication-dependent histone H3/H4 chaperone complex CAF-1, CHAF1A and CHAF1B, as top candidates in the screen (Fig. 2B,C) and their validation in secondary assays prompted us to investigate a potential role of the CAF-1 complex in preventing the mislocalization of CENP-A. As we observed a slightly greater effect on the intensity of YFP–CENP-A following depletion of CHAF1B than CHAF1A (Fig. 2C; median Z-scores: CHAF1B, 4.99; CHAF1A, 3.28), we tested the function of CHAF1B in regulating CENP-A localization. For these experiments, we used the well-characterized HeLa^CENP-A–TAP^ cell line, which stably overexpresses 4- to 5-fold higher levels of CENP-A tagged with tandem affinity purification (TAP) tags compared to those of endogenous CENP-A in parental HeLa cells (Nechemia-Arbely et al., 2019). Using four independent siRNAs and an siRNA pool targeting *CHAF1B*, we validated the depletion of the CHAF1B protein in HeLa^CENP-A–TAP^ cells (Table S1) (Fig.  S1A). No significant effect on the levels of CHAF1A or RbAp48 proteins were observed in CHAF1B-depleted cells (Fig. S1B).

**Fig. 3.**
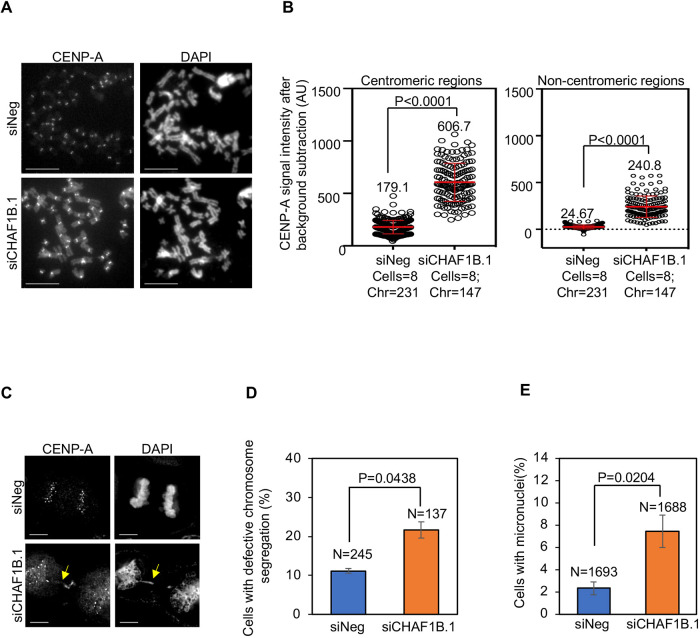
**CHAF1B prevents the mislocalization of CENP-A to non-centromeric regions and CIN phenotypes.** (A) Representative images of metaphase chromosome spreads showing the localization of CENP-A at centromeric and non-centromeric regions in HeLa^CENP-A–TAP^ cells transfected with the indicated siRNAs. Metaphase chromosome spreads were prepared 72 h post transfection, and cells were immunostained using an antibody against CENP-A and stained with DAPI. Scale bars: 5 µm. (B) Quantification of CENP-A signal intensities at centromeric (left) and non-centromeric (right) regions in metaphase chromosome spreads of HeLa^CENP-A–TAP^ cells transfected with the indicated siRNAs. Each circle represents a spot on a centromeric or non-centromeric region. ‘Cells’ and ‘Chr’ denote the numbers of cells and chromosomes analyzed, respectively. The red lines indicate mean±s.d. for YFP signal intensities across areas measured in the number of cells indicated from three independent experiments. A.U., arbitrary units. (C) Representative images showing chromosome segregation status in HeLa^CENP-A–TAP^ cells transfected with the indicated siRNAs. The yellow arrow indicates lagging chromosomes. Scale bars: 5 µm. (D,E) The proportion of cells exhibiting defective chromosome segregation (D) and cells with micronuclei (E) in HeLa^CENP-A–TAP^ cells transfected with the indicated siRNAs. *N* denotes the number of cells analyzed. Error bars represent the s.e.m. from three independent experiments. *P*-values shown in B,D,E were calculated using unpaired, two-tailed *t*-test.

We next examined whether the increased nuclear signal of YFP–CENP-A upon depletion of CHAF1B in the imaging-based assay of YFP nuclear intensity is due to the mislocalization of CENP-A to non-centromeric regions. To this end, metaphase chromosome spreads were prepared from HeLa^CENP-A–TAP^ cells transfected with either negative-control siRNA (siNeg) or siCHAF1B.1 and immunostained with anti-CENP-A to visualize CENP-A localization (Fig. 3A; Fig. S2). Centromeric regions were defined as the constriction site in a chromosome with the brightest signal of CENP-A, whereas the non-centromeric regions were defined as any region other than centromeres within the chromosome arms. Based on these criteria, we observed the mislocalization of CENP-A at non-centromeric regions in CHAF1B-depleted HeLa^CENP-A–TAP^ cells (Fig. 3A; Fig. S2A). Quantitative analysis of CENP-A signal intensities showed elevated levels of CENP-A at centromeric and non-centromeric regions upon depletion of CHAF1B (Fig. 3B).

As mislocalization of overexpressed CENP-A contributes to CIN in HIRA-depleted cells (Fig. 1D,E), we assessed whether the depletion of CHAF1B also leads to CIN in these cells. Analysis of fixed cells immunostained with the anti-CENP-A antibody and stained with DAPI showed that CHAF1B-depleted cells exhibited a significant increase in the proportion of cells with evidence of defective chromosome segregation (e.g. lagging chromosomes, uncongressed chromosomes and DNA bridges) (Fig. 3C,D). CHAF1B-depleted cells also exhibited a higher incidence of micronuclei (Fig. 3E). These results show that CHAF1B prevents mislocalization of CENP-A and CIN.

**Fig. 4.**
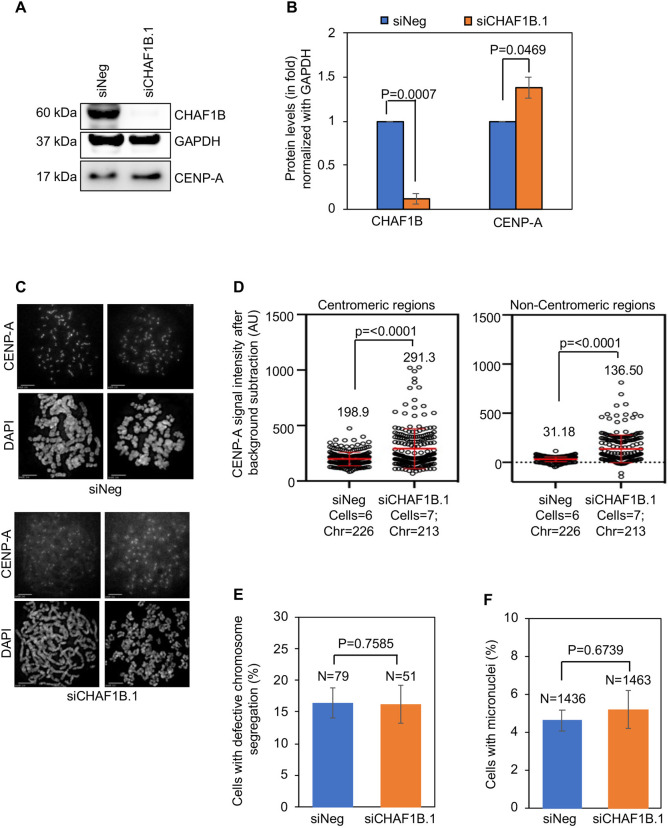
**Endogenous CENP-A is mislocalized to non-centromeric regions in parental HeLa cells depleted of CHAF1B.** (A) Western blots of lysates prepared from parental HeLa cells transfected with the indicated siRNAs for 72 h and analyzed using antibodies as indicated. (B) Quantification of CHAF1B and endogenous CENP-A levels in whole-cell extracts of parental HeLa cells transfected with the indicated siRNAs. The protein levels were normalized against those of GAPDH and expressed as fold increase or decrease relative to those of control cells. (C) Representative images of metaphase chromosome spreads showing the localization of CENP-A at centromeric and non-centromeric regions in parental HeLa cells transfected with the indicated siRNAs. Metaphase chromosome spreads were prepared 72 h post transfection, and cells were immunostained with an antibody against CENP-A and stained with DAPI. Scale bars: 5 µm. (D) Quantification of CENP-A signal intensities at centromeric (left) and non-centromeric (right) regions in metaphase chromosome spreads of parental HeLa cells transfected with the indicated siRNAs. Each circle represents a spot on a centromeric or non-centromeric region. ‘Cells’ and ‘Chr’ denote the numbers of cells and chromosomes analyzed, respectively. Red lines indicated mean±s.d. for YFP signal intensities across areas measured in the number of cells indicated from two independent experiments. (E,F) The proportion of cells exhibiting defective chromosome segregation (E) and cells with micronuclei (F) in parental HeLa cells transfected with the indicated siRNAs. *N* denotes the number of cells analyzed. Error bars in B,E,F represent s.e.m. from four (B) and three (E,F) independent experiments. *P*-values shown in B,D–F were calculated using unpaired, two-tailed *t*-test in GraphPad Prism 9.

We next examined whether endogenous CENP-A exhibits altered localization upon CHAF1B depletion using parental HeLa cells that do not overexpress CENP-A. Western blot analysis confirmed the depletion of CHAF1B in parental HeLa cells transfected with siCHAF1B.1 (Fig. 4A,B). Additionally, we observed elevated levels of endogenous CENP-A in CHAF1B-depleted whole-cell extracts (Fig. 4A,B). In agreement with increased CENP-A levels seen by western blotting, quantitative analysis of CENP-A signal intensities on metaphase chromosomes revealed significant, 1.4-fold higher levels of CENP-A at centromeric regions and 4.3-fold higher levels of CENP-A at non-centromeric regions in CHAF1B-depleted cells compared to those in control cells (Fig. 4C,D). Importantly, the 4.3-fold increase in non-centromeric CENP-A observed in CHAF1B-depleted parental HeLa cells was lower than the 9.7-fold increase observed in CHAF1B-depleted HeLa^CENP-A–TAP^ cells (Fig. 3B). Analysis of the CIN phenotypes upon depletion of CHAF1B in parental HeLa cells showed no significant increase in the proportion of cells with defective chromosome segregation (Fig. 4E) or incidence of micronuclei (Fig. 4F). These results are consistent with our previous observations for a positive correlation of CIN to the levels of CENP-A mislocalization (Shrestha et al., 2017). We conclude that CENP-A is mislocalized in parental HeLa and HeLa^CENP-A–TAP^ cells depleted for CHAF1B and that increased levels of mislocalized CENP-A contribute to CIN phenotypes upon CHAF1B depletion in HeLa^CENP-A–TAP^ cells.

### Genome-wide analysis shows mislocalization of CENP-A in CHAF1B-depleted cells

To further assess the function of CHAF1B in regulating the chromosomal localization of CENP-A, we used CUT&RUN sequencing to map the distribution of CENP-A in asynchronous populations of HeLa^CENP-A–TAP^ cells transfected with siNeg or siCHAF1B.1. Flow cytometry analysis was used to assess cell cycle progression in control or CHAF1B-depleted cells. The qualitative analysis showed similar cell cycle profiles in HeLa^CENP-A–TAP^ cells transfected with siNeg or siCHAF1B.1 (Fig. S3A). However, quantification showed a slight reduction in the proportion of G1 cells, without significant changes in S and G2/M populations upon CHAF1B depletion, compared to those of control cells (Fig. S3B). As fluorescence-activated cell sorting (FACS) is based on analysis of 2N/4N DNA content, we also examined the expression of cell cycle-regulated proteins and nuclear morphology to assess cell cycle progression. Western blot analysis of lysates prepared from HeLa^CENP-A–TAP^ cells transfected with siNeg or siCHAF1B.1 showed no differences in the levels of the cell cycle-regulated proteins cyclin D2 (CCND2), cyclin E1 (CCNE1) and phosphorylated histone H3 (pH3), which exhibit increased levels in G1, S and mitotic cell cycle stages, respectively (Fig. S3C,D). Furthermore, using DAPI staining for nuclear morphology, cells were scored in interphase, prometaphase, metaphase, anaphase and cytokinesis. Our results showed no significant change in the proportion of cells in a particular cell cycle stage upon CHAF1B depletion compared to that of control cells (Fig. S3E). Based on these data, we conclude that CHAF1B depletion does not affect cell cycle progression.

**Fig. 5.**
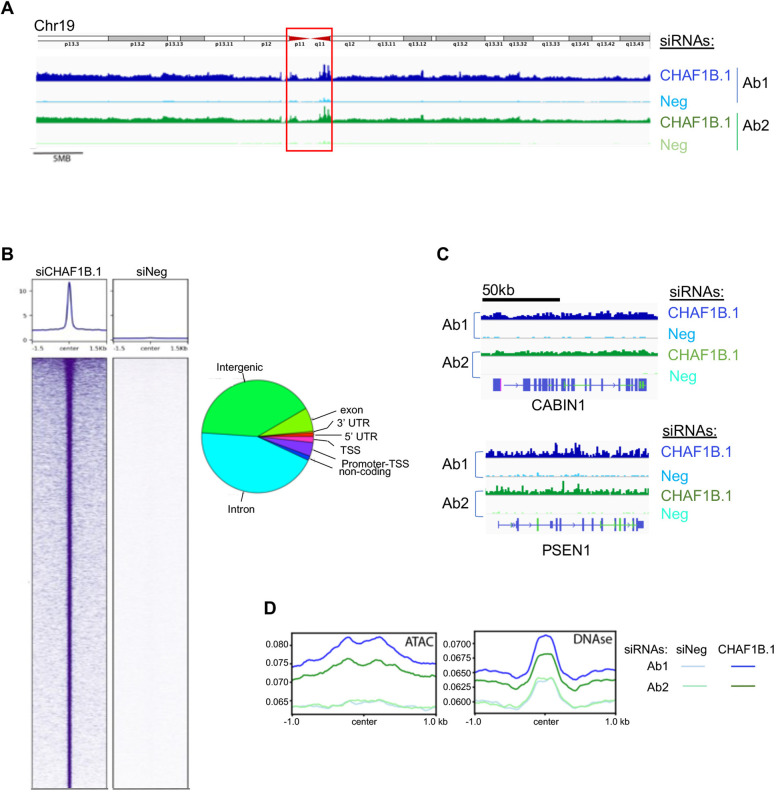
**Genome-wide analysis of CUT&RUN sequencing shows the mislocalization of CENP-A upon depletion of CHAF1B in HeLa^CENP-A–TAP^ cells.** (A) CUT&RUN sequencing of TAP-tagged CENP-A showing the distribution of CENP-A across chromosome 19 (CHM13v2) in asynchronous HeLa^CENP-A–TAP^ cells transfected with the indicated siRNAs for 72 h. The analysis was performed using two different antibodies against CENP-A. An ideogram of chromosome 19 is shown above the CUT&RUN sequencing tracks and the red box indicates the endogenous centromere. (B) Peaks of CENP-A identified by CUT&RUN sequencing in CHAF1B-depleted cells are shown relative to those of siNeg-transfected control cells. The pie chart shows the distribution of CENP-A peaks across various genomic regions. TSS, transcription start site; UTR, untranslated region. (C) Representative distributions of CENP-A at the gene level are shown for two genes: *PSEN1* (Chr14) and *CABIN1* (Chr22). (D) Overlap of CENP-A CUT&RUN profile from HeLa^CENP-A–TAP^ cells transfected with the indicated siRNAs plotted against previously identified ATAC-seq peaks (GSM2830382; Cho et al., 2018) and DNase I-hypersensitive sites (GSM763533; Mercer et al., 2013).

The results of the genome-wide CUT&RUN sequencing performed using two different CENP-A antibodies showed that the depletion of CHAF1B leads to increased deposition of CENP-A across the entire length of all chromosomes (Fig. S4A,B). We observed enrichment of CENP-A at endogenous centromeric α-satellite repeats in both control and CHAF1B-depleted cells, as indicated by the representative results for chromosome 19 shown in Fig. 5A (red box). These results are consistent with CENP-A occupying a fraction of α-satellite repeats under conditions of normal endogenous expression. Despite mapping the CUT&RUN sequences to the telomere-to-telomere (T2T) assembly of the human genome, it is likely that the ambiguity in uniquely mapping short read sequences to the α-satellite repeats might influence peak detection at the centromere, but nonetheless indicates higher levels of CENP-A at α-satellite repeats.

Although peak-calling approaches do not encompass all the CENP-A within a chromosome, CENP-A accumulation in peaks identified by model-based analysis for ChIP-Seq (MACS2) revealed a distribution of CENP-A across all regions of the genome at proportions that reflect genomic organization (Fig. 5B). CENP-A peaks were also observed within regulatory, intronic and exonic regions as demonstrated by the distribution of CENP-A at two loci, *CABIN1* and *PSEN1* (Fig. 5C). Consistent with mislocalization of CENP-A to open chromatin such as assay for transposase-accessible chromatin (ATAC)-accessible sites and DNase-hypersensitive regions (Athwal et al., 2015; Lacoste et al., 2014), we observed increased levels of CENP-A at these regions upon CHAF1B depletion (Fig. 5D). Collectively, our cell biology-based analysis and genome-wide approaches demonstrate that CHAF1B prevents mislocalization of overexpressed CENP-A to non-centromeric regions for chromosomal stability.

### Depletion of CHAF1B contributes to the enrichment of CENP-A in chromatin

**Fig. 6.**
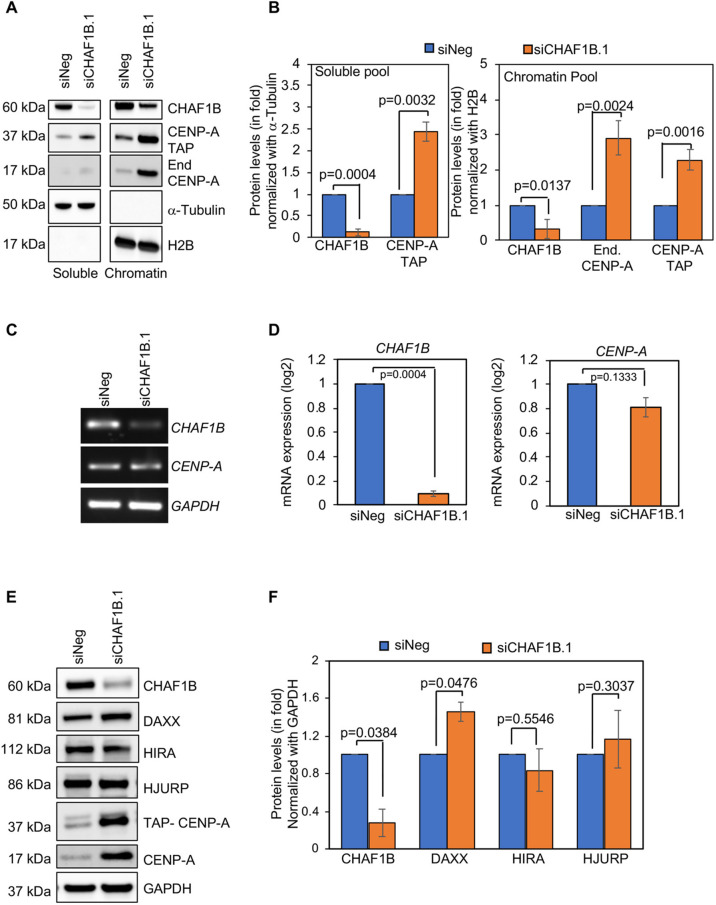
**CHAF1B-depleted cells show an enrichment of CENP-A in chromatin and increased levels of the histone H3.3 chaperone DAXX.** (A) Western blots of cellular fractionated lysates prepared from HeLa^YFP–CENP-A-low^ cells transfected with the indicted siRNAs for 72 h and analyzed using antibodies as indicated. (B) Quantification of CHAF1B, TAP-tagged CENP-A and endogenous (‘End.’) CENP-A in the soluble (left) and chromatin (right) fractions of HeLa^CENP-A–TAP^ cells transfected with the indicated siRNAs. The protein levels were normalized against those of α-tubulin and H2B for the soluble and chromatin fractions, respectively, and expressed as fold increase or decrease relative to those of control cells. (C,D) Gels (C) and bar charts (D) from semi-quantitative RT-PCR and RT-qPCR, respectively showing the mRNA levels of *CHAF1B* and *CENPA* in HeLa^CENP-A–TAP^ cells transfected with the indicated siRNAs. *GAPDH* was used as loading control. In D, the levels were normalized against those of *GAPDH* and expressed as a log_2_ fold change. *DAXX* mRNA levels were also analyzed as part of these experiments (Fig. S5A,B), and the data for *CHAF1B* and *GAPDH* are shown again in Fig. S5A,B for comparison with the *DAXX* results. (E,F) Western blot analysis (E) and quantification (F) of lysates prepared from HeLa^CENP-A–TAP^ cells transfected with the indicated siRNAs for 72 h and analyzed using antibodies as indicated. GAPDH was used as a loading control and used to normalize the levels of other proteins to express as a fold increase or decreased relative to those of control cells. For B,D,F, error bars represent s.e.m. from three independent experiments and *P*-values were calculated using unpaired, two-tailed *t*-test.

Studies with budding yeast have shown that mislocalization of Cse4 correlates with the enrichment of Cse4 in chromatin (Au et al., 2008; Hewawasam et al., 2010; Ohkuni et al., 2018). The mislocalization of CENP-A in CHAF1B-depleted cells (Fig. 3A) led us to examine whether CENP-A is enriched in the chromatin fraction of these cells. We examined the levels of endogenous and TAP-tagged CENP-A in the soluble and chromatin fractions from HeLa^CENP-A–TAP^ cells transfected with either siNeg or siCHAF1B.1. Western blot analysis of α-tubulin and histone H2B confirmed the efficacy of our fractionation protocol (Fig. 6A). Following depletion of CHAF1B, a reduction in CHAF1B was observed in both the soluble and chromatin fractions (Fig. 6A,B). CHAF1B-depleted cells showed enrichment of endogenous and TAP-tagged CENP-A in the chromatin fraction compared to that in control cells (Fig. 6A,B). The soluble fraction showed enrichment of TAP-tagged CENP-A following depletion of CHAF1B (Fig. 6A,B). Elevated levels of CENP-A were consistent with the observation in CHAF1B-depleted parental HeLa cells (Fig. 4A,B). To examine whether higher levels of CENP-A protein were due to higher transcription of *CENPA*, we used real-time quantitative PCR (RT-qPCR) to quantify mRNA levels of *CENPA* and *CHAF1B* using primers listed in Table S1. As expected, mRNA levels of *CHAF1B* were significantly lower in CHAF1B-depleted cells; however, in these cells, the mRNA levels of *CENPA* were not significantly altered compared to those in control cells (Fig. 6C,D). We conclude that the higher levels of the CENP-A protein in CHAF1B-depleted cells are not due to altered transcription of *CENP-A*. In addition, our data for increased levels of chromatin-associated CENP-A correlates with the increased mislocalization of CENP-A to non-centromeric regions upon CHAF1B depletion (Fig. 3).

### CHAF1B-depleted cells exhibit increased expression of the histone H3.3 chaperone DAXX

The mislocalization of CENP-A in CHAF1B-depleted HeLa^CENP-A–TAP^ cells prompted us to examine whether this observation was due to the altered expression of histone chaperones that regulate the localization of CENP-A. For example, HJURP is a CENP-A-specific chaperone that recruits CENP-A to centromeres, and a balance between the levels of HJURP and CENP-A prevents CENP-A mislocalization and CIN (Nye et al., 2018). DAXX is a histone H3.3 chaperone that promotes mislocalization of overexpressed CENP-A in HeLa cells (Lacoste et al., 2014; Shrestha et al., 2017). HIRA, as described earlier, is a replication-independent histone H3 chaperone that prevents mislocalization of overexpressed CENP-A (Ciftci-Yilmaz et al., 2018; Lacoste et al., 2014; Nye et al., 2018). Western blot analysis showed that the levels of DAXX, but not of HJURP or HIRA, were increased upon CHAF1B depletion in HeLa^CENP-A–TAP^ compared to those in control cells (Fig. 6E,F). Consistent with our results in Fig. 6A,B, the levels of CENP-A–TAP and endogenous CENP-A were higher in CHAF1B-depleted cells. Interestingly, a recent study reported the increased transcription of DAXX in CHAF1B-depleted Burkitt lymphoma cells (Zhang et al., 2020), an observation we confirmed in HeLa^CENP-A–TAP^ cells using RT-qPCR (Fig. S5A,B).

### Depletion of DAXX suppresses the mislocalization of CENP-A and CIN in CHAF1B-depleted cells

The depletion of DAXX suppresses the mislocalization of CENP-A, CIN phenotypes and the cell invasion phenotype in HeLa and DLD1 cells overexpressing CENP-A (Lacoste et al., 2014; Shrestha et al., 2017, 2021). These studies and our results for increased levels of DAXX (Fig. 6E,F) led us to examine whether DAXX contributes to the mislocalization of CENP-A in CHAF1B-depleted HeLa^CENP-A–TAP^ cells. To this end, we co-depleted CHAF1B and DAXX in HeLa^CENP-A–TAP^ cells. Western blot analysis confirmed depletion of CHAF1B and DAXX in cells transfected with siCHAF1B.1 and/or siDAXX (Fig. S6A,B). Consistent with the results presented in Fig. 6, we observed an enrichment of endogenous and TAP-tagged CENP-A in CHAF1B-depleted cells (Fig. S6A,B). DAXX depletion alone did not affect CENP-A levels significantly; however, cells co-depleted of DAXX and CHAF1B exhibited reduced levels of endogenous and TAP-tagged CENP-A compared to those in cells depleted of CHAF1B alone (Fig. S6A,B). These results show that DAXX contributes to the higher levels of CENP-A in CHAF1B-depleted HeLa^CENP-A–TAP^ cells.

Based on the positive correlation of higher levels of CENP-A to its mislocalization, we hypothesized that depletion of DAXX would suppress the mislocalization of CENP-A in CHAF1B-depleted HeLa^CENP-A–TAP^ cells. We examined the localization of CENP-A in interphase cells depleted of DAXX with or without CHAF1B co-depletion by immunostaining with an anti-CENP-A antibody. Analysis of the nuclear CENP-A signal showed that the co-depletion of CHAF1B and DAXX reduced the percentage of cells exhibiting CENP-A mislocalization compared to that of cells depleted for CHAF1B (Fig. S6C,D). Depletion of DAXX alone did not affect the nuclear localization of CENP-A (Fig. S6C,D). Based on these results, we conclude that DAXX contributes to increased nuclear localization of CENP-A in CHAF1B-depleted interphase cells.

**Fig. 7.**
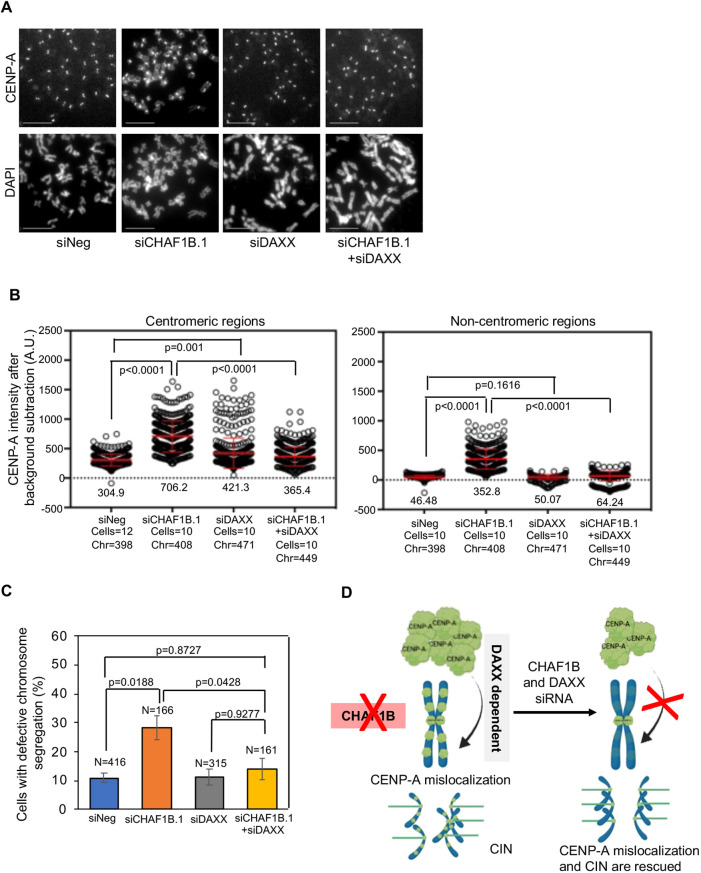
**DAXX contributes to the mislocalization of CENP-A and CIN in CHAF1B-depleted cells.** (A) Representative images of metaphase chromosome spreads showing the localization of CENP-A at centromeric and non-centromeric regions in HeLa^CENP-A–TAP^ cells transfected with the indicated siRNAs for 72 h. Metaphase chromosome spreads were prepared 72 h post transfection, and cells were immunostained with an antibody against CENP-A and stained with DAPI. Scale bars: 5 µm. (B) Quantification of CENP-A signal intensities at centromeric (left) and non-centromeric (right) regions in metaphase chromosome spreads of HeLa^CENP-A–TAP^ cells transfected with the indicated siRNAs. Each circle represents a spot on a centromeric or non-centromeric region. ‘Cells’ and ‘Chr’ denote the numbers of cells and chromosomes analyzed, respectively. Red lines indicated mean±s.d. for YFP signal intensities across areas measured in the number of cells indicated from three independent experiments. (C) The proportion of cells exhibiting defective chromosome segregation in HeLa^CENP-A–TAP^ cells transfected with the indicated siRNAs. *N* denotes the number of cells analyzed. Error bars represent s.e.m. from three independent experiments. *P*-values shown in B,C were calculated using one way-ANOVA with Tukey's post hoc test. (D) Model for CENP-A mislocalization and CIN in CHAF1B-depleted cells. We propose that depletion of CHAF1B promotes DAXX-mediated mislocalization of CENP-A to non-centromeric regions. Support for this model includes the CUT&RUN sequencing data and the suppression of CENP-A mislocalization and CIN phenotypes by depletion of DAXX in CHAF1B-depleted cells.

We next prepared metaphase chromosome spreads to examine CENP-A localization at centromeric and non-centromeric regions in HeLa^CENP-A–TAP^ cells transfected with either siNeg or siCHAF1B.1, and/or siDAXX. As expected, we observed the mislocalization of CENP-A at non-centromeric regions in CHAF1B-depleted HeLa^CENP-A–TAP^ cells but not in cells depleted for DAXX alone. However, cells co-depleted for CHAF1B and DAXX showed reduced mislocalization of CENP-A at non-centromeric regions (Fig. 7A; Fig. S6E). Quantitative analysis showed significantly reduced levels of CENP-A at centromeric and non-centromeric regions upon depletion of DAXX in CHAF1B-depleted cells (Fig. 7B). Based on these observations, we conclude that the mislocalization of CENP-A in CHAF1B-depleted cells is DAXX dependent. If this is the case, we hypothesized that a reduction in DAXX expression should suppress the chromosome segregation defects observed in CHAF1B-depleted HeLa^CENP-A–TAP^ cells. Consistent with this hypothesis, co-depletion of CHAF1B and DAXX significantly reduced the proportion of cells exhibiting defective chromosome segregation compared to that of CHAF1B-depleted cells. The proportion of cells with defective chromosome segregation upon DAXX depletion was comparable to that observed in control cells (Fig. 7C). We conclude that DAXX contributes to the mislocalization of CENP-A and CIN phenotypes in CHAF1B-depleted cells.

## DISCUSSION

The overexpression and mislocalization of CENP-A is observed in many cancers and this correlates with poor prognosis (Saha et al., 2020; Smith and Sheltzer, 2022; Wang et al., 2021; Xu et al., 2020; Zhang et al., 2016). Hence, it is critical to define the causes and consequences of CENP-A mislocalization. We previously showed that mislocalization of overexpressed CENP-A contributes to CIN in HeLa and DLD1 cells and in a xenograft tumor model (Shrestha et al., 2021). In this study, we used an imaging-based high-throughput RNAi screen to identify proteins that prevent the mislocalization of CENP-A. Validation and characterization experiments focusing on one of the top factors identified in our screen, CHAF1B, defined a function for this histone H3/H4 chaperone in preventing the mislocalization of CENP-A and CIN.

The identification of two subunits of the CAF-1 complex (CHAF1A and CHAF1B) in our screen suggested a critical role for this complex in preventing the mislocalization of CENP-A. The primary role of the CAF-1 complex is to deposit newly synthesized H3/H4 heterodimers into chromatin during DNA synthesis (reviewed in Volk and Crispino, 2015). It has been shown that in the absence of CAF-1 function, canonical H3.1/H3.2 deposition is perturbed, and that the replication-independent histone chaperone HIRA compensates these effects by depositing the histone variant H3.3 to maintain chromatin integrity (Gomes et al., 2019; Ray-Gallet et al., 2011). Our data suggest that non-canonical mislocalization of CENP-A might serve as an alternative mechanism to compensate for the global defect in the deposition of H3.1/H3.2 in CHAF1B-depleted cells. Support for this hypothesis is based on the following phenotypes in CHAF1B-depleted cells: (1) increased mislocalization of CENP-A to non-centromeric regions on mitotic chromosomes (Fig. 3A,B); (2) Higher levels of chromatin-associated CENP-A (Fig. 6A,B, Fig. 7D); and (3) genome-wide mislocalization of CENP-A to non-centromeric regions based on CUT&RUN experiments (Fig. 5). Mislocalization of CENP-A was also observed upon depletion of CHAF1B in parental HeLa cells without overexpression of CENP-A. Most importantly, we defined the physiological consequence for CENP-A mislocalization by showing that CHAF1B-depleted cells exhibit CIN phenotypes with defects in chromosome segregation and increased incidence of micronuclei. We conclude that CHAF1B contributes to chromosomal stability in CENP-A-overexpressing cells.

Cell biology approaches provide evidence for CENP-A mislocalization to non-centromeric regions upon depletion of CHAF1B (Figs 3 and 4). Consistent with these results, a genome-wide approach, CUT&RUN sequencing, with asynchronous cells revealed a broad distribution of mislocalized CENP-A across the genome (Fig. S4). Enrichment of CENP-A at highly accessible regions as defined by either DNase I hypersensitivity or ATAC-accessible sequences was observed in CHAF1B-depleted cells (Fig. 5D). These results are consistent with other studies using multiple cell lines, including SW480, HeLa, and HuRef human lymphoblastoids, showing the mislocalization of overexpressed CENP-A to promoters or enhancers of highly expressed genes and at CTCF-binding sites, DNase I-hypersensitive regions and ATAC-accessible chromatin sites, which have been identified by the Encyclopedia of DNA Elements (ENCODE) project and are functionally related to transcriptional activity (Athwal et al., 2015; Nechemia-Arbely et al., 2019; Hayden et al., 2013; Lacoste et al., 2014). A recent genome-wide localization study revealed the association of CENP-A with transcriptionally active sites along chromosome arms in the early G1 phase of the cell cycle and an enrichment of CENP-A at DNase I-hypersensitive sites was more apparent in G1 cells (Nechemia-Arbely et al., 2019). Based on these results, the authors proposed that DNA replication facilitates the removal of ectopic CENP-A with localization restricted to centromeric chromatin in mitotic cells (Nechemia-Arbely et al., 2019). This raises the possibility that enrichment of CENP-A at DNase I-hypersensitive and ATAC-accessible sites observed in CHAF1B-depleted cells could be due to accumulation of cells in the G1–S transition. However, flow cytometry analysis, western blotting for cell cycle markers and nuclear morphology analysis showed that CHAF1B depletion did not delay the G1–S transition (Fig. S3), and this was consistent with a previous study that did not observe cell cycle delay upon depletion of CHAF1B in HeLa cells (Nabatiyan and Krude, 2004). We propose that CENP-A mislocalization compensates for global defects in canonical H3.1/H3.2 deposition in CHAF1B-depleted HeLa cells.

Previous studies have implicated roles of CAF-1 in chromosomal localization of CENP-A in different model organisms. For example, in budding yeast, CAF-1 binds to centromeric chromatin throughout the cell cycle and has been shown to promote the localization of overexpressed Cse4 to centromeric and non-centromeric chromatin (Hewawasam et al., 2018; Ohkuni et al., 2020). We have shown that the replication-independent histone chaperone HIR complex prevents mislocalization of overexpressed Cse4 and chromosome loss (Ciftci-Yilmaz et al., 2018). Studies have shown that combined loss of CAF-1 and HIR functions leads to mislocalization of Cse4 (Lopes da Rosa et al., 2011) and defects in centromeric structure and kinetochore function (Sharp et al., 2002). In *Drosophila*, Mis16, a homolog of RbAp48, interacts with CENP-A and this interaction is important for the centromeric localization of CENP-A (Demirdizen et al., 2019; Furuyama et al., 2006). In human cells, although RbAp48 interacts with CENP-A, depletion of RbAp48 alone does not affect the centromeric localization of CENP-A (Hayashi et al., 2004). However, recent evidence showing interaction of human CAF-1 components with CENP-A suggests that the CAF-1 complex might promote CENP-A loading post DNA replication (Nechemia-Arbely et al., 2019). Nevertheless, we did not observe reduced centromeric CENP-A signals in mitotic cells either in parental cells with no overexpression of CENP-A (Fig. 4) or in HeLa^CENP-A–TAP^ with overexpression of CENP-A (Fig. 3) upon CHAF1B depletion, suggesting that CHAF1B depletion alone might not reduce centromeric CENP-A loading.

We next investigated the molecular basis for the mislocalization of CENP-A upon depletion of CHAF1B. Given that DAXX contributes to the mislocalization of overexpressed CENP-A in HeLa cells (Lacoste et al., 2014; Shrestha et al., 2017), we examined whether the mislocalization of CENP-A in CHAF1B-depleted cells was dependent on DAXX. Our results showed that the mRNA and protein levels of DAXX were increased in CHAF1B-depleted cells (Fig. 6E; Fig. S5); comparable results have been reported in Burkitt lymphoma cells (Zhang et al., 2020). Depletion of DAXX in CHAF1B-depleted cells suppressed both CENP-A mislocalization and CIN (Fig. 7A,B). Based on these findings, we conclude that depletion of CHAF1B promotes CENP-A mislocalization in a DAXX-dependent pathway (Fig. 7D).

In conclusion, we identified several potential regulators of CENP-A localization and defined a novel role of the histone H3/H4 chaperone CHAF1B in preventing CENP-A mislocalization and CIN. Mechanistically, we showed that DAXX contributes to CENP-A mislocalization and CIN in CHAF1B-depleted cells. These observations are potentially relevant from a clinical standpoint, as the Catalogue of Somatic Mutations in Cancer (COSMIC) reveals several mutations in CHAF1B that are related to human malignancies and as 80% of these mutations are non-functional missense mutations (Forbes et al., 2015). To this end, it will be of interest to examine whether missense mutations in CHAF1B contribute to CENP-A mislocalization and CIN phenotypes. Additionally, a recent study suggests that inducers of metastasis can suppress CAF-1 function and promote chromatin alterations that favor the expression of metastatic genes (Gomes et al., 2019). In summary, our studies show that proper deposition of canonical histones by histone chaperones such as CHAF1B prevents CIN and that defects in these pathways might contribute to aneuploidy in CENP-A-overexpressing cancers.

## MATERIALS AND METHODS

### Cell culture

All cell lines were cultured at 37°C with 5% CO_2_ supply in Dulbecco's modified Eagle's medium (DMEM; 12491023, Thermo Fisher Scientific) supplemented with 10% fetal calf serum (FCS; F6178-500ml, Sigma-Aldrich), penicillin/streptomycin (15140122, Thermo Fisher Scientific), fungizone (15290018, Thermo Fisher Scientific) and L-glutamine (A2916801, Thermo Fisher Scientific). For frozen stocks, cells were mixed in freezing medium (DMEM with 50% FCS and 5% DMSO) and stored at −80°C. All cell lines were tested for mycoplasma-free status using the Universal Mycoplasma Detection Kit [30-1012K, American Type Culture Collection (ATCC)] according to the manufacturer's instructions. The generation of the HeLa^YFP–CENP-A-low^, HeLa^YFP–CENP-A-high^ and HeLa^CENP-A–TAP^ cells was described previously (Shrestha et al., 2017; Nechemia-Arbely et al., 2019). The parental HeLa cell line was purchased from ATCC (CCL-2).

### siRNA library preparation for high-throughput imaging screen

The Silencer Select Epigenetics synthetic siRNA library (A30085, Thermo Fisher Scientific) was received in lyophilized form in 96-well plates (0.25 nM synthesis scale). The library contains three independent siRNAs oligonucleotides per gene (one siRNA per well) for each of the 521 targeted human genes. Each siRNA in the library was resuspended in 50 µl water (5 µM final concentration) using a Janus liquid handler (PerkinElmer), frozen at −20°C overnight, and then thawed to increase siRNA solubility. The resuspended siRNAs oligonucleotides were transferred and compressed from a 96- to a 384-well plate format using a Janus liquid handler to obtain a complete ‘mother’ copy of the library. The Janus liquid handler was used to transfer 10 µl of undiluted siRNA solution from a mother plate (5 µM final concentration) to a ‘daughter’ 384-well LDV acoustic liquid handler compatible plate (Beckman Coulter). Scrambled negative-control siRNA (siNeg.1) was added to column 23 (odd rows), whereas positive biological siRNA controls were added to column 24 (siHIRA.2 in odd rows and siHIRA.3 in even rows) of each daughter plate. Daughter plates were used as a source to generate 384-well image-ready plates (CellCarrier Ultra 384, 6057802, PerkinElmer) by spotting 150 µl of the diluted siRNA at the bottom of an empty plate using an Echo 525 acoustic liquid handler (Beckman Coulter). The arrayed siRNAs oligonucleotides were aseptically dried for 30 min in a laminar flow hood. Dried plates were sealed and stored at −20°C until the day of the transfection.

### siRNA library transfection and fluorescence staining

On the day of the transfection, frozen imaging assay-ready plates were thawed, equilibrated at room temperature, and centrifuged at 500 ***g*** for 5 min. Then, 20 µl of OPTIMEM (31985088, Thermo Fisher Scientific) containing 50 nl of RNAiMax transfection reagent (13778075, Thermo Fisher Scientific) was added to each well of the imaging plate and incubated for 30 min at room temperature. Next, 20 µl of trypsinized and resuspended HeLa^YFP–CENP-A-high^ cells in DMEM containing 20% FCS was added at a concentration of 1500 cells/well. Plates were incubated at room temperature for 30 min and then at 37°C for 72 h. The final concentration of each siRNA was 20 nM. At 72 h post transfection of the siRNA, cells were fixed in paraformaldehyde (PFA; 28908, Thermo Fisher Scientific) for 15 min at room temperature by adding 40 µl per well of 8% PFA in PBS directly to the medium using a Bluewasher plate washer/dispenser (BlueCatBio). The same instrument was used to wash plates in 50 µl/well of PBS and to stain with 50 µl/well of DAPI (0.5 µg/ml). Plates were stored at 4°C until imaged. Two independent biological replicates of the screen were performed.

### Automated image acquisition and high-content image analysis

The Yokogawa CV7000S high-throughput spinning-disk microscope was used for automated imaging of stained 384-well plates. Briefly, 405 nm and 488 nm excitation lasers were used for DAPI and YFP–CENP-A detection, respectively. Both channels also used a 405/488/561/640 nm excitation dichroic mirror and a 40× air lens (NA 0.95). Images were sequentially acquired on a single plane using a 16-bit sCMOS camera (2560×2160 pixel, 2×2 binning, pixel size: 0.325 μm) and 445/45 nm or 525/50 nm bandpass filters for the DAPI and YFP–CENP-A channels, respectively. Images were corrected with proprietary Yokogawa software to compensate for camera background and illumination artifacts, and then saved as .tiff files.

For high-content image analysis, image files were imported in the Columbus 2.7.1 image storage and analysis software (PerkinElmer). A Columbus image analysis pipeline was used to measure YFP–CENP-A nuclear levels upon transfection of the siRNA library. The DAPI image was first used for nuclear segmentation and then we measured mean fluorescence intensity in the YFP–CENP-A channel over each of the nuclear regions of interest (ROIs). Nuclei with a roundness value below 0.775, mostly corresponding to segmentation artifacts, and nuclei touching the border of the image were excluded from the calculation. The average of the mean YFP–CENP-A nuclear fluorescence intensity across all the cells in the well was calculated on a per-well basis. Well-level results were exported in tabular format as .txt files.

### siRNA screen statistical analysis

We used R (https://www.R-project.org/) and the Bioconductor cellHTS2 package (Boutros et al., 2006) for the statistical analysis of the screen. Briefly, per-well mean aggregated image analysis results for the different cellular parameters were used as the raw input for the cellHTS2 analysis. The data were retained on the additive scale and not log transformed. Additionally, the raw well measurements on a per-plate basis were normalized using the B-score normalization method with per-plate variance adjustment using the median of the library on the plate as the centrality parameter for the normalization. The B-scores for all the wells in a biological replicate were scored using the Z-score method. Each siRNA oligonucleotide was run in two biological replicates (each one producing a single ‘well-level Z-score’). The final Z-score for each siRNA – siRNA-level Z-score – was obtained by averaging the Z-scores for each of the two biological replicates (wells). The median Z-score was calculated on a per-gene basis. Each gene in the library was targeted by three individual siRNA oligonucleotides. The ‘gene-level Z-score’ was obtained by calculating the median of the three ‘siRNA-level Z-scores’ for that gene. As the library had three siRNAs per gene, the median Z-score corresponds to the siRNA with the second strongest biological effect in the assay on a per-gene basis. The gene-level Z-score was used to rank the effect of siRNA-mediated gene silencing. Ranking siRNAs with the median Z-score allowed us to prioritize genes whose silencing showed a biological effect with at least two independent siRNAs. This approach reduces but does not eliminate false-positive genes in the list identified because of off-target effects.

### RNAi validation and characterization studies

All siRNA transfections were performed using Lipofectamine RNAiMax (13778075, Thermo Fisher Scientific) according to the manufacturer's instructions. For RNAi, siRNA oligonucleotides and the transfecting reagent were diluted in Opti-MEM reduced serum medium (31985088, Thermo Fisher Scientific). The sequences and associated information for all siRNAs used are provided in Table S1.

### Chromosome spread preparation and immunofluorescence

For CENP-A localization studies, chromosome spreads were prepared as follows: after growth to 70% confluency, cells were treated with 200 ng/ml colcemide (10295892001, Roche) for 3–5 h. Cells were centrifuged at 500 ***g*** for 5 min at room temperature in 15 ml Falcon tubes and cell pellets were resuspended in 1 ml of hypotonic solution (75 mM KCl) added dropwise, mixed and incubated at 37°C for 15 min. Cells were counted and resuspended to a concentration of 120,000 cells/ml. Next, 250 µl of the cell suspension was centrifuged in the cytospin at 100 ***g*** for 5 min. Cells were then hydrated with KCM buffer (10 mM Tris-HCl pH 8.0, 120 mM KCl, 20 mM NaCl, 0.5 mM EDTA and 0.1% Triton X-100) for 2 min, followed by permeabilization with KCM buffer containing 0.5% Triton X-100 for 10 min. Cells were blocked with KCM containing 2% bovine serum albumin (BSA) for 30 min and incubated with mouse anti-CENP-A antibody (ADI-KAM-CC006-E, Enzo Life Sciences) as the primary antibody for 1 h at room temperature, washed three times in KCM buffer, incubated with goat anti-mouse DY 488 (35502, Thermo Fisher Scientific) as the secondary antibody, and washed three times in KCM buffer. Both antibodies were diluted in KCM buffer containing 2% BSA at 1:500 dilution. Cells were then fixed with 3.7% paraformaldehyde (28908, Thermo Fisher Scientific) in KCM buffer for 10 min and washed with PBS, stained with DAPI for 10 min, washed with PBS containing 0.1% Triton X-100, followed by a PBS wash and a final wash with water, and mounted on a slide.

### Immunostaining and immunoblotting

For immunostaining, cells were fixed with ice-cold methanol for 1 min, followed by blocking with 1% BSA in PBS containing 0.1% Tween-20 (PBST) for 45 min at room temperature. Cells were incubated in primary antibodies for 1 h at room temperature, washed three times in PBST, and incubated with secondary antibodies and DAPI for 1 h at room temperature. After three washes with PBST, cells were mounted on slides using Prolong gold antifade mounting medium containing DAPI (P36935, Thermo Fisher scientific). Mouse anti-CENP-A (ADI-KAM-CC006-E, Enzo Life Sciences) as the primary antibody and goat anti-mouse DY 488 (35502, Thermo Fisher Scientific) as the secondary antibody were used at 1:500 dilution in 1% BSA in PBST.

For western blot analysis, primary antibodies as indicated in the figure legends were used at the indicated dilutions as follows: rabbit anti-HIRA (ab129169, Abcam), mouse anti-CENP-A (ADI-KAM-CC006-E, Enzo Life Sciences), rabbit anti-CHAF1B (ab109442, Abcam), anti-CHAF1A (ab126625, Abcam) and anti-RbAp48 (ab1765, Abcam) at 1:500 dilution; mouse anti-α-tubulin (ab176560, Abcam), rabbit anti-DAXX (25C12, Cell Signaling Technology), rabbit anti-cyclin E1 (20808, Cell Signaling Technology), mouse anti-cyclin D2 (sc-376676, Santa Cruz Biotechnology) and rabbit anti-HJURP (HPA008436, Sigma-Aldrich) at 1:1000 dilution; and mouse anti-GAPDH (MA5-15738, Invitrogen) and rabbit anti-H2B (ab1790, Abcam) at 1:2000 dilution. HRP-conjugated secondary antibodies against mouse (GENA931, Sigma-Aldrich) and rabbit (GENA934, Sigma-Aldrich) were used at 1:4000 dilution. Blots were treated with SuperSignal West Pico PLUS Chemiluminescent substrate (34578, Thermo Fisher Scientific) prior to imaging using a Bio-Rad Imager. Fiji was used to quantify signal intensities of bands from western blots and expressed as fold increase or decrease relative to the control bands. Full blot images for blots shown in this paper are presented in Fig. S7.

### Microscopy and image analysis

Immunostained cells were imaged on a DeltaVision Core system (Applied Precision/GE Healthcare, Issaquah, WA, USA) consisting of an Olympus IX70 inverted microscope (Olympus America, Melville, NY, USA) with a 100× NA 1.4 oil immersion objective and a CoolSnap HQ 12-bit camera (Photometrics, Tucson, AZ, USA) controlled by softWoRx software. Filters used for imaging were FITC (excitation 490/20 nm; emission 528/38 nm), RD-TR (excitation 555/28 nm; emission 617/73 nm) and DAPI (excitation 360/40 nm; emission 457/50 nm) of the 86,000 Sedat Quadruple Filter Set (Chroma Technology, Bellows Falls, VT, USA). *Z*-stacks of at least ten focal planes were acquired with an exposure of 0.1–0.5 s, depending on the filter. Signal intensity was measured using the data inspector tool in *softWoRx*. To prepare the figures, images were deconvolved, unless otherwise mentioned, with softWoRx and scaled manually to 8 bit using a linear lookup table (LUT) and the same range of scaling for all the images.

### Quantitative immunofluorescence analysis

To calculate fluorescence intensities, boxes of 8×8 pixels were drawn on centromeric regions as ascertained by bright foci of CENP-A and on non-centromeric region as ascertained by the signal outside the centromeric region on a chromosome (chromosome spreads). For background correction, four boxes of 8×8 pixels were drawn at four random areas within the cytoplasm in the same cell. The maximum intensity values from all drawn areas were obtained using the data inspector tool in softWoRx*.* The final fluorescence intensity for each protein was calculated by subtracting the average background intensity. Intensity measurements were done for at least ten centromeric and non-centromeric spots in each cell for an average of ten cells from two or three independent experiments. For statistical analysis, average values from more than 100 centromeric or non-centromeric spots were calculated and used as a mean to calculate s.e.m. across areas measured.

### Histone extraction protocol

Cells were harvested and washed twice with ice-cold PBS and resuspended in Triton extraction buffer [TEB; PBS containing 0.5% Triton-X 100 (v/v), 2 mM phenylmethylsulfonyl fluoride (PMSF), 0.02% (w/v) NaN_3_] at a cell density of 10^7^ cells per ml. Cells were lysed on ice for 10 min with gentle stirring, centrifuged at 6500 ***g*** for 10 min at 4°C, the supernatant (soluble pool) was transferred to a new tube and the pellet was saved as the chromatin fraction. The pellet was washed in half the volume of TEB and centrifuged at 6500 ***g*** for 10 min at 4°C, the supernatant discarded, and the chromatin pellet resuspended in 0.2 N HCl at a density of 4×10^7^ nuclei per ml and incubated with 0.2 N HCl overnight at 4°C to obtain the acid-extracted histones. Samples were centrifuged the next day at 6500 ***g*** for 10 min at 4°C to pellet the debris and the supernatant was saved as acid-extracted histones. HCl was neutralized with 2 M NaOH at 1/10 of the volume of the supernatant. Protein concentration was determined using the Bradford assay.

### Flow cytometry analysis

Cells were harvested by trypsinization and centrifuged at 500 ***g*** for 5 min. Cell pellets were washed twice with PBS and fixed in 70% ethanol for 30 min at 4°C. Fixed cells were then washed twice with PBS followed by 20 µg/ml of RNaseA treatment for 30 min at room temperature. Propidium iodide was added to the cell suspension to a final concentration of 5 µg/ml. Flow cytometric analysis was carried out on a CytoFLEX S (Beckman Coulter) using CytExpert and data were processed using FlowJo (version 10.8.1).

### CUT&RUN sequencing

CENP-A CUT&RUN sequencing was performed as previously described, with modifications (Hogan et al., 2021). Briefly, for each CUT&RUN sequencing experiment, ∼4×10^5^ siRNA-transfected HeLa^CENP-A–TAP^ cells were harvested. HeLa^CENP-A–TAP^ cells were incubated with concanavalin A-coated magnetic beads (BioMag), permeabilized in digitonin buffer (20 mM HEPES-NaOH pH 7.5, 150 mM NaCl, 0.5 mM spermidine, 0.0025% digitonin and 1× Roche protease inhibitor cocktail). Following permeabilization of the bead–cell slurry, bead–cells were incubated with anti-CENP-A antibodies (1:50, 07-574, Millipore; or 1:50, MA1-20832, Invitrogen), anti-H3K27me3 (1:50, 9733, Cell Signaling Technology) or IgG as a control [1:50 in antibody buffer (20 mM HEPES-NaOH pH 7.5, 150 mM NaCl, 0.5 mM spermidine, 0.0025% digitonin, 2 mM EDTA, 0.1% BSA, 100 nM trichostatin A, 0.1 U/ml citrate synthase, 1 mM oxaloacetic acid and 1× Roche protease inhibitor cocktail)] overnight at 4°C. Following two washes in digitonin buffer, beads were incubated in 50 μl digitonin buffer with 1× CUTANA pAG-MNase (EpiCypher, 15-1016) for 1 h at 4°C. Following a 30 min incubation with ice-cold Ca^2+^ incubation buffer (3.5 mM HEPES-NaOH pH 7.5, 10 mM CaCl_2_ and 0.0025% digitonin), the beads were resuspended with EGTA-stop buffer [170 mM NaCl, 20 mM EGTA, 0.0025% digitonin, 50 μg/ml RNase A, 50 pg/ml CUTANA *E. coli* Spike-in DNA (EpiCypher 18-1401)] and incubated for 30 min at 37°C. Library preparation was performed as previously described (Hogan et al., 2021). Libraries were sequenced on the Illumina HiSeq 2X150 with 1% PhiX control V3 library (derived from the small, well-characterized bacteriophage genome; FC-110-3001, Illumina) as a control. Reads were trimmed to generate 50 bp paired-end reads. Reads were aligned to the CHM13v2 and hg38 genomes using the CETO pipeline (Elizabeth Bartom, Northwestern University; https://github.com/ebartom/NGSbartom) and MACS2 (Zhang et al., 2008) to identify CENP-A peaks. Bigwig files from the alignment to CHM13v2 were normalized using *E. coli* spike-in using Bamtools (Meers et al., 2019). CUT&RUN signal tracks were visualized using IGV2.12 (https://software.broadinstitute.org/software/igv/2.12.x). The genomic distribution of CENP-A peaks was determined using the ChipSeeker R package (Yu et al., 2015). Previously published ATAC-seq and DNase I hypersensitivity data used in this study are available under the accession numbers GSM2830382 and GSM763533, and are displayed on hg38 aligned bigwig files using DeepTools (Ramírez et al., 2016).

### Statistical analysis

*P*-values were calculated using the unpaired two-tailed *t*-test or one way-ANOVA with Tukey's post hoc test as indicated in the figure legends using GraphPad Prism 9.

## Supplementary Material

10.1242/joces.260944_sup1 Supplementary information
