# Supplementary material for: The histone H3/H4 chaperone CHAF1B prevents the mislocalization of CENP-A for chromosomal stability
Source: J Cell Sci. 2023 May 31;136(10):jcs260944. doi: 10.1242/jcs.260944 (PMC10281518; doi:10.1242/jcs.260944)
Supplement: Supplementary information [file joces-136-260944-s1.pdf]

Fig. S1.

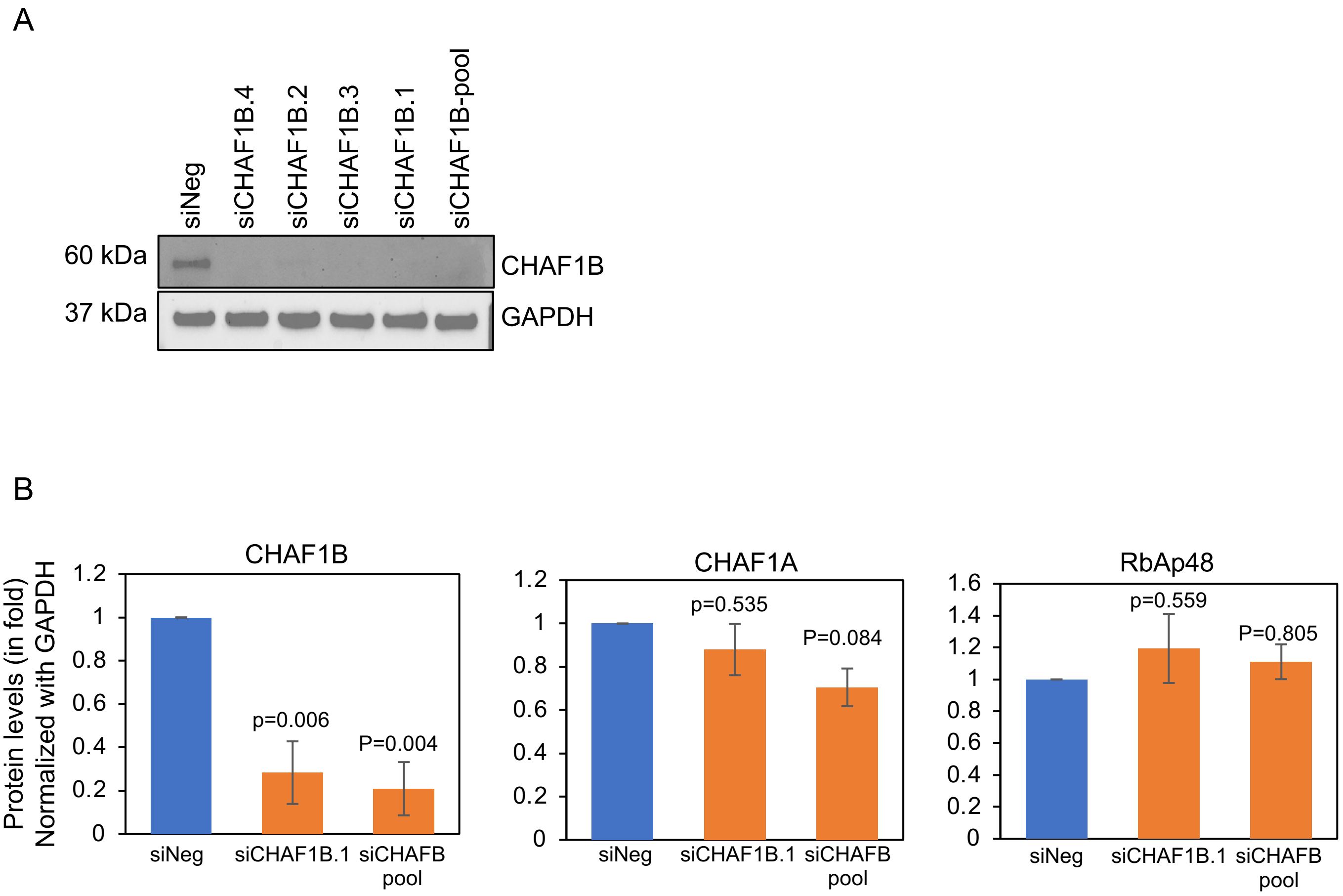

Fig. S1. CHAF1B depletion does not affect the protein levels of other components of the CAF-1 complex. (A) Western blots of lysates prepared from HeLa$^{CENP\text{-}A\text{-}TAP}$ cells transfected with the indicated siRNA oligos for 72 hours and analyzed using antibodies as indicated. GAPDH was used as a loading control. (B) Blots quantifying levels of proteins indicated on top of each graph in HeLa$^{CENP\text{-}A\text{-}TAP}$ cells transfected with the indicated siRNA oligos for 72 hours. Levels were normalized against GAPDH and expressed as fold increase or decrease relative to control cells. Error bar represents the s.e.m. from three independent experiments. P values were calculated using one way-ANOVA test.

Journal of Cell Science • Supplementary information

Fig. S2.

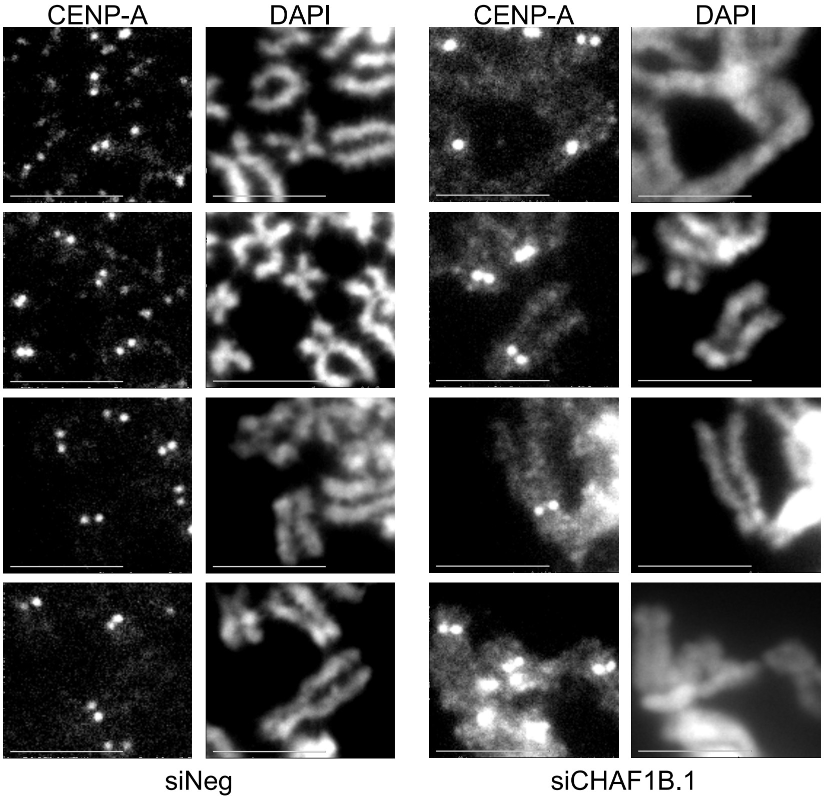

Fig. S2. CENP-A mislocalization to non-centromeric regions in CHAF1B-depleted HeLa$^{\text{CENP-A-TAP}}$ cells. Metaphase chromosome spread images showing localization of CENP-A at centromeric and non-centromeric regions in HeLa$^{\text{CENP-A-TAP}}$ cells transfected with the indicated siRNA oligos for 72 hours. Metaphase chromosome spreads were prepared post 72 hours siRNA transfection and cells were immunostained with antibody against CENP-A and stained with DAPI. Chromosomes were zoomed to 4x from the normal scale. Scale bar: 1 µm.

Fig. S3.

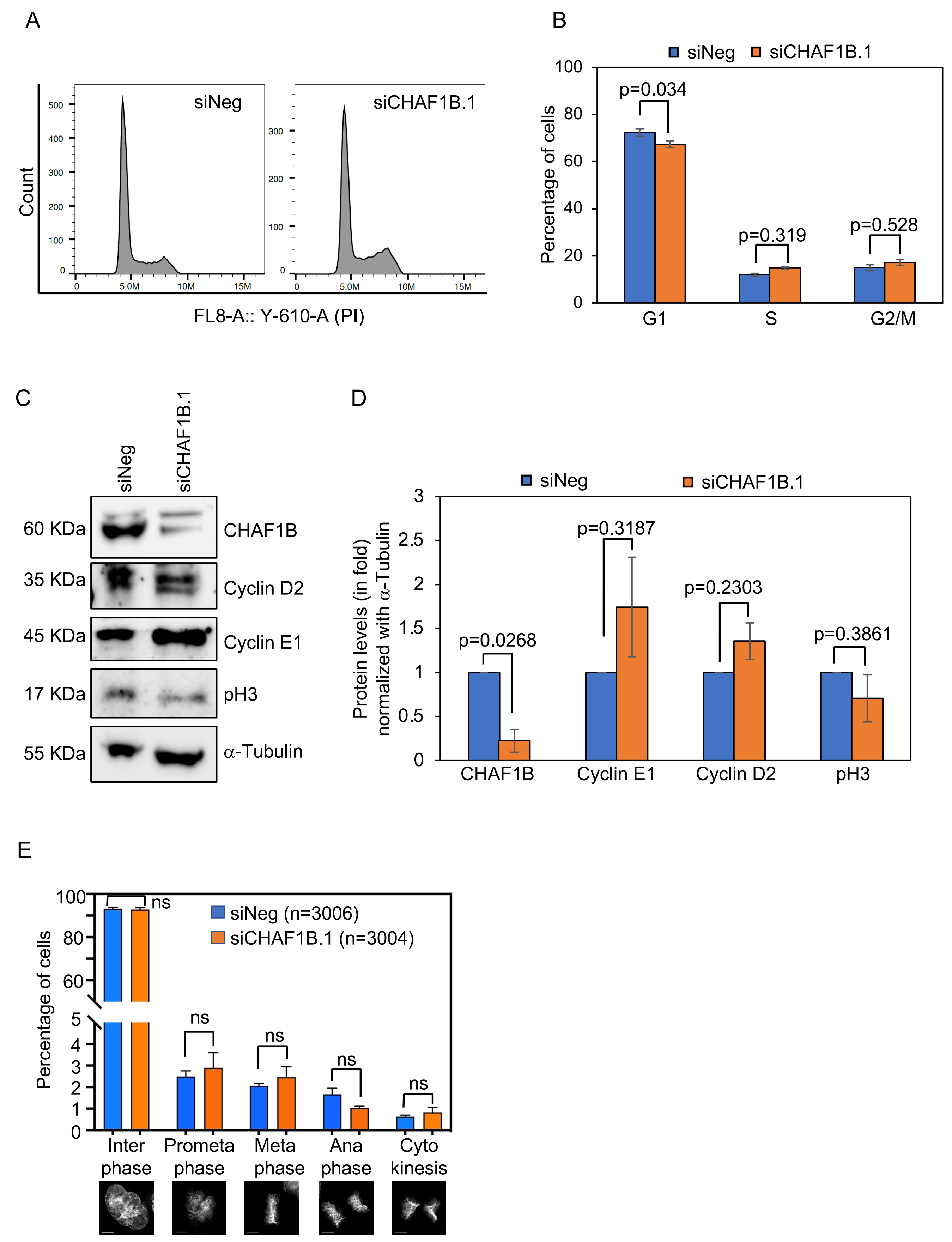

Journal of Cell Science • Supplementary information

Fig. S3. Depletion of CHAF1B does not contribute to defects in cell cycle progression. (A-B) Flow cytometry profile (A) and quantification (B) for cell cycle stages of HeLa$^{CENP\text{-}A\text{-}TAP}$ cells transfected with the indicated siRNA oligos for 72 hours. Cells were stained with Propidium Iodide (PI) for flow cytometry analysis. (C-D) Western blots (C) and quantification (D) of lysates prepared from HeLa$^{CENP\text{-}A\text{-}TAP}$ cells transfected with the indicated siRNA oligos for 72 hours and analyzed using antibodies as indicated. Cyclin D2, Cyclin E1 and pH3 were used as markers for G1, S and mitotic stages, respectively. α-Tubulin was used as a loading control and used to normalize the levels of other proteins to express as fold increase or decrease relative to control cells. (E) Bar charts showing the proportion of cells in different cell cycle stages as indicated in HeLa$^{CENP\text{-}A\text{-}TAP}$ cells transfected with the indicated siRNA oligos for 72 hours. Lower panel shows the representative images of cells stained with DAPI in different cell cycle stages. For B, D and E, error bars represent s.e.m. from three independent experiments. p values shown were calculated using Two- way Anova test (for B) and Unpaired, two-sided t-test (for D and E). “n” denotes number of cells analyzed from three independent experiments.

Journal of Cell Science • Supplementary information

Fig. S4.

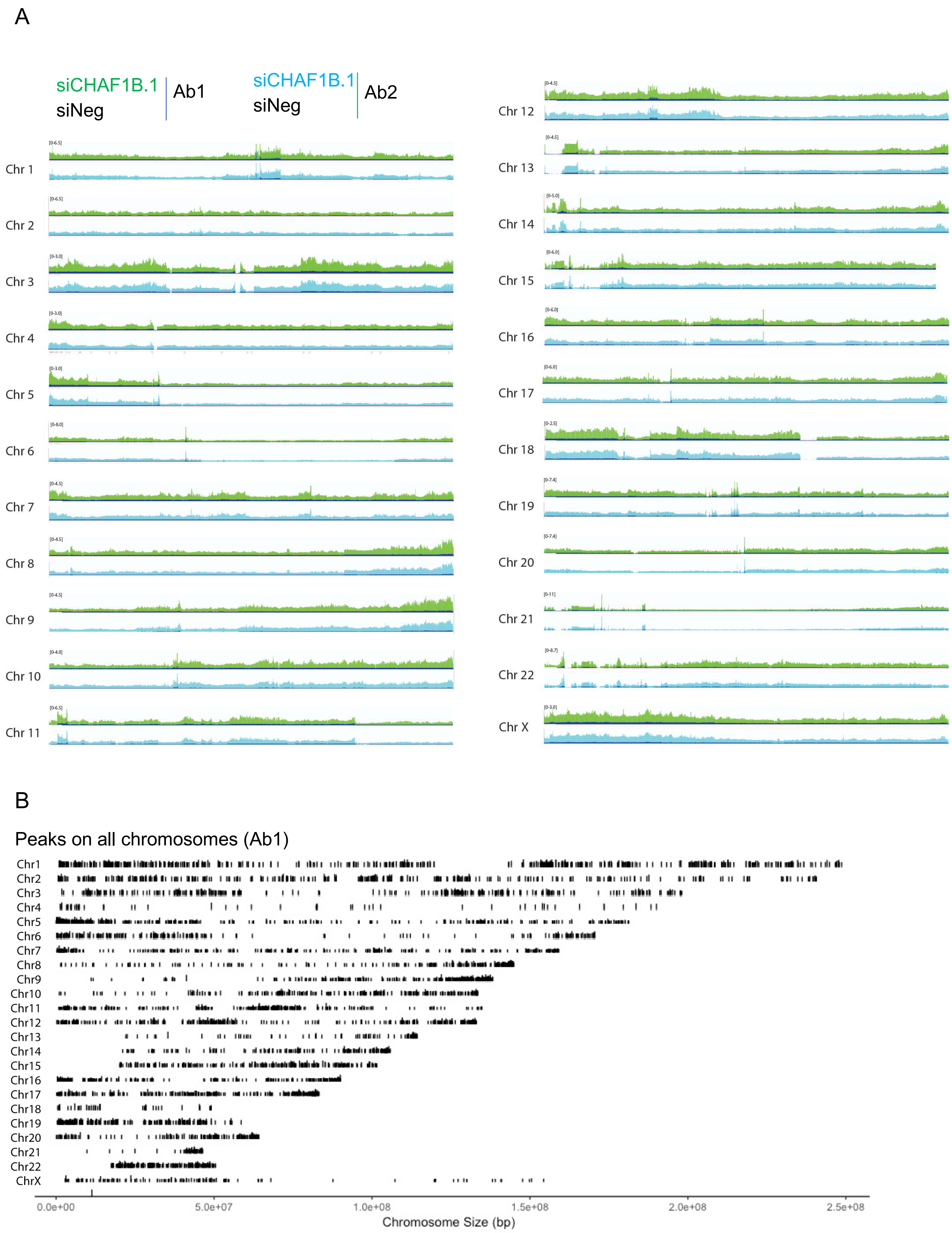

Journal of Cell Science • Supplementary information

Fig. S4. Genome-wide analysis using CUT&RUN sequencing shows the mislocalization of CENP-A upon depletion of CHAF1B across all chromosomes. (A) Pattern of CENP-A occupancy from CUT&RUN sequencing done in asynchronous HeLa$^{CENP\text{-}A\text{-}TAP}$ cells transfected with siNeg (black) or siCHAF1B.1 (green or blue). Two independent antibodies against CENP-A were used. (B) Peaks of CENP-A identified by Model-based Analysis for ChIP-Seq (MACS2) in each track in CHAF1B-depleted cells as identified by one CENP-A antibody.

Fig. S5.

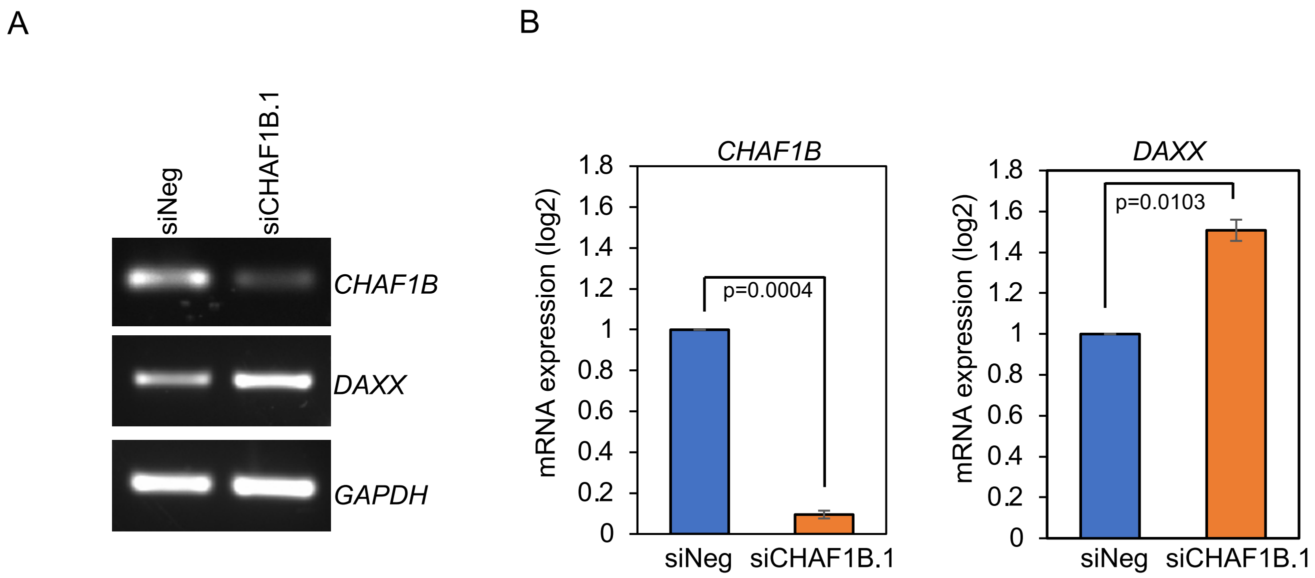

Fig. S5. Increased transcription of *DAXX* in CHAF1B-depleted cells. (A-B) Gels (A) and bar charts (B) from semi quantitative RT-PCR and RT-qPCR, respectively, showing the mRNA levels of *CHAF1B* and *DAXX* in HeLa$^{CENP\text{-}A\text{-}TAP}$ cells transfected with the indicated siRNA oligos for 72 hours. *GAPDH* was used as loading control. In B, the levels were normalized against *GAPDH* and expressed as a log fold-change. Because analysis of *DAXX* mRNA was included in the same experiments as in Fig. 6C and D, the data for *CHAF1B* and *GAPDH* were used from the data set shown in Fig. 6C,D. Error bars represent the s.e.m. from three independent experiments and p-values were calculated using Unpaired two-sided t-test.

Journal of Cell Science • Supplementary information

Fig. S6.

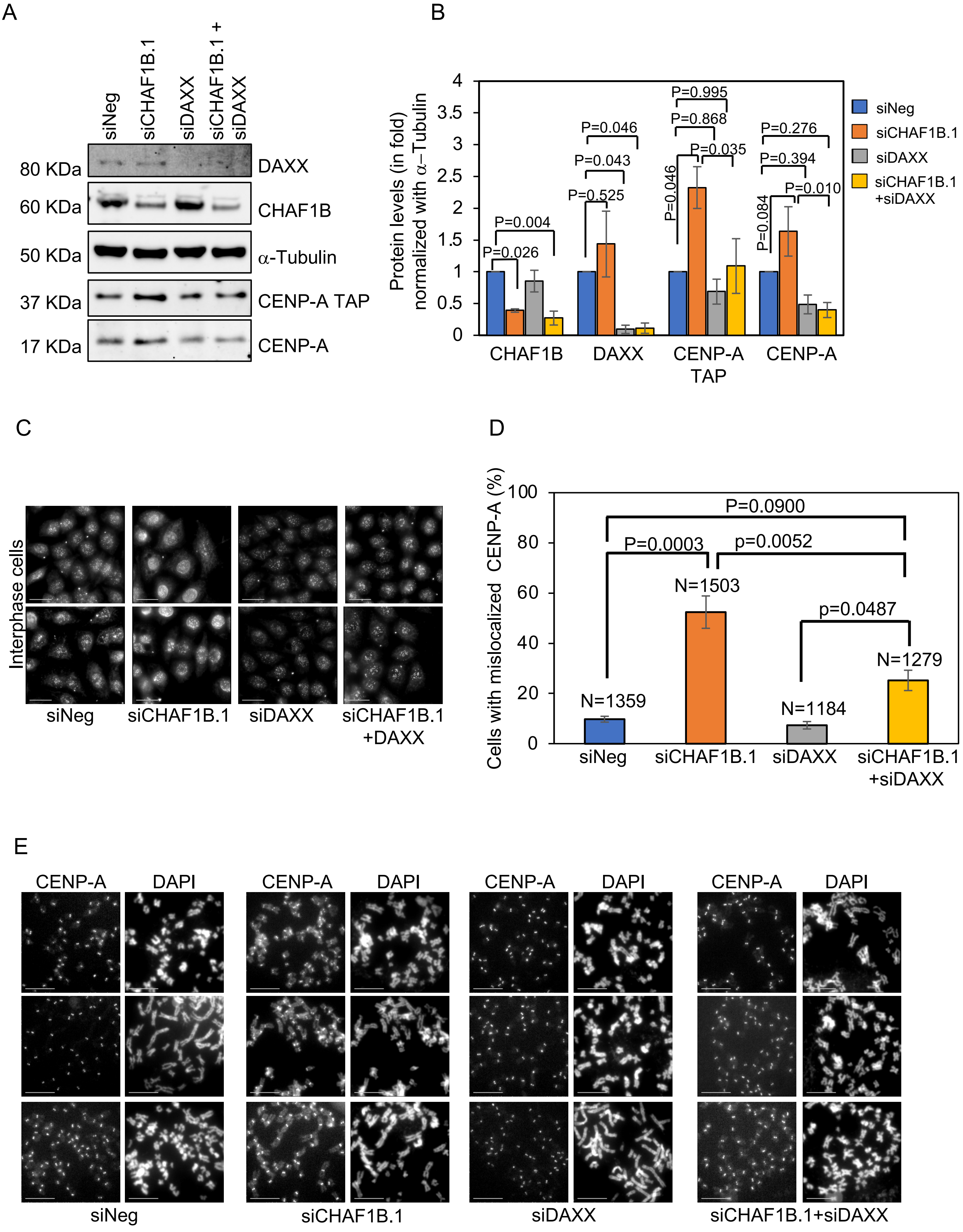

Journal of Cell Science • Supplementary information

Fig. S6. DAXX depletion suppresses the mislocalization of CENP-A in CHAF1B-depleted cells. (A-B) Western blots (A) and quantification (B) of lysates prepared from HeLa$^{CENP\text{-}A\text{-}TAP}$ cells transfected with the indicated siRNA oligos for 72 hours and analyzed using antibodies as indicated. α-Tubulin was used as a loading control and used to normalize the levels of other proteins to express as fold increase or decrease relative to control cells. (C) Immunostained images showing the nuclear localization of CENP-A in interphase cell stages of HeLa$^{CENP\text{-}A\text{-}TAP}$ cells transfected with the indicated siRNA oligos for 72 hours. Cells were fixed and immunostained with antibody against CENP-A. Scale bar: 15 µm. (D) Bar chart showing the proportion of cells with CENP-A mislocalization in HeLa$^{CENP\text{-}A\text{-}TAP}$ transfected with the indicated siRNA oligos. For B and D, error bars represent the s.e.m. from three independent experiments and p values were calculated using one way- ANOVA test. "N" in D denotes number of cells analyzed in three experiments. (E) Metaphase chromosome spread images showing localization of CENP-A at centromeric and non-centromeric regions in HeLa$^{CENP\text{-}A\text{-}TAP}$ cells transfected with the indicated siRNA oligos for 72 hours. Metaphase chromosome spreads were prepared, and cells were immunostained with antibody against CENP-A and stained with DAPI. Scale bar: 5 µm.

Fig. S7A.

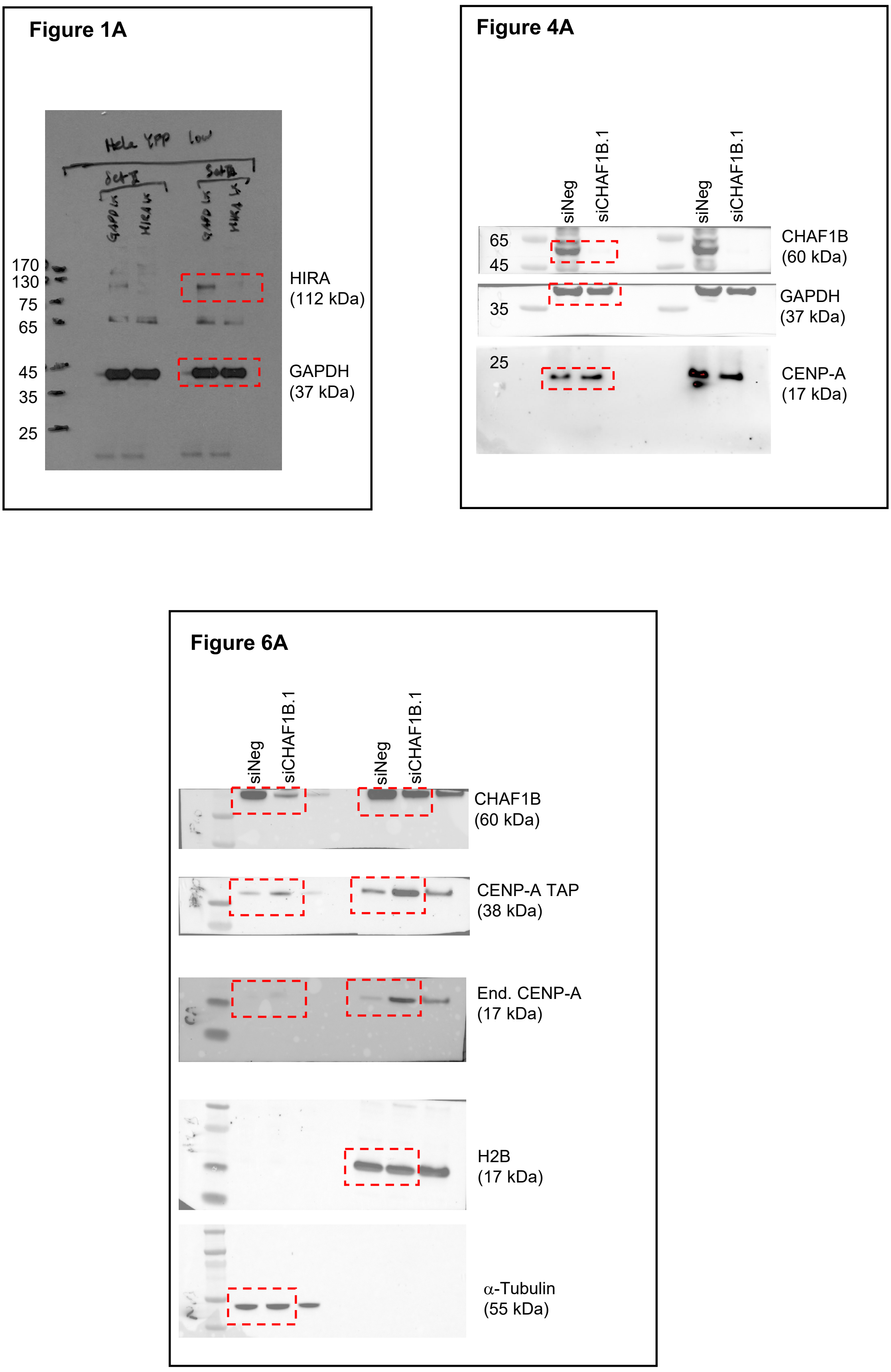

Journal of Cell Science • Supplementary information

Fig. S7B

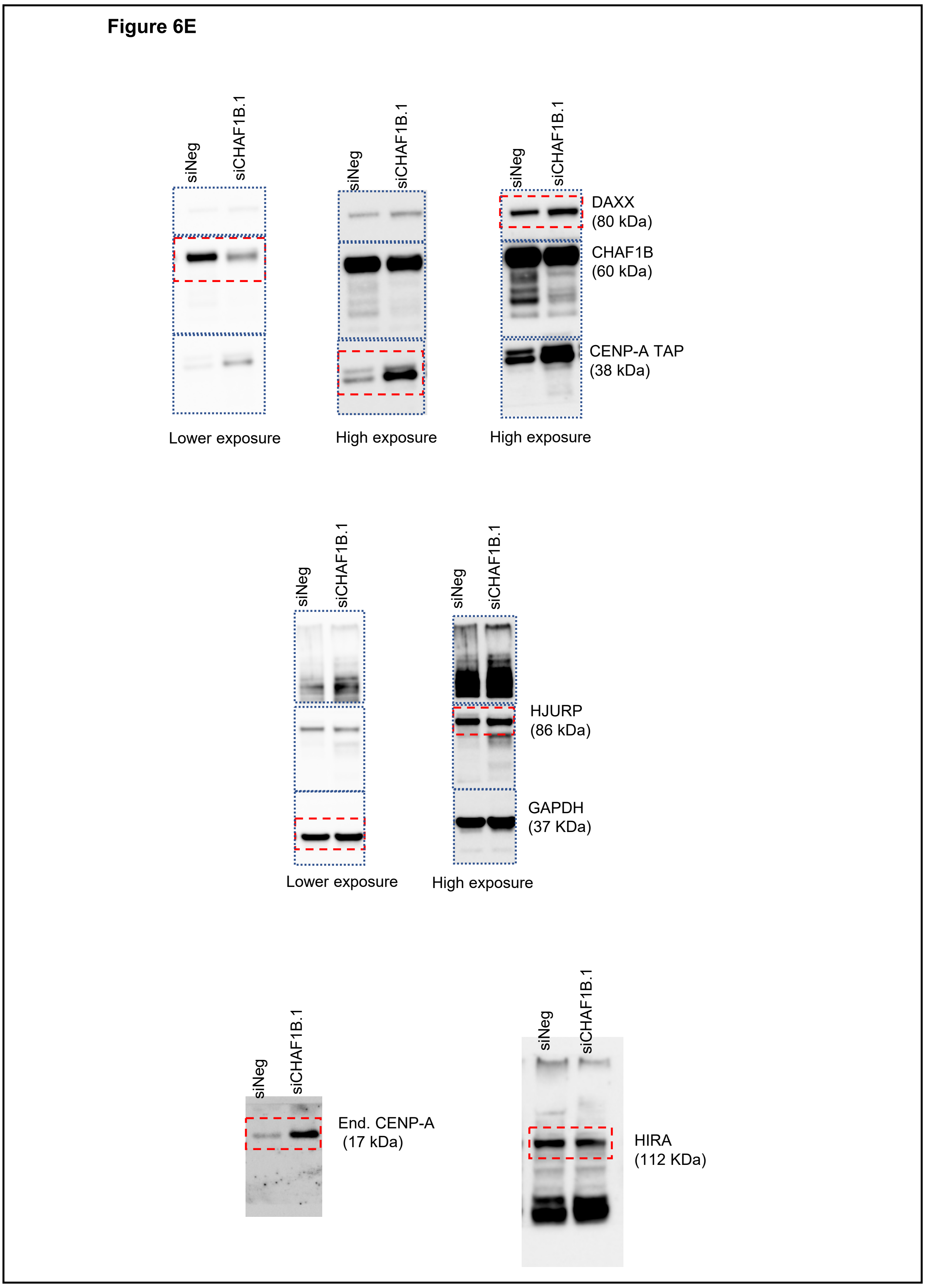

Journal of Cell Science • Supplementary information

Fig. S7C

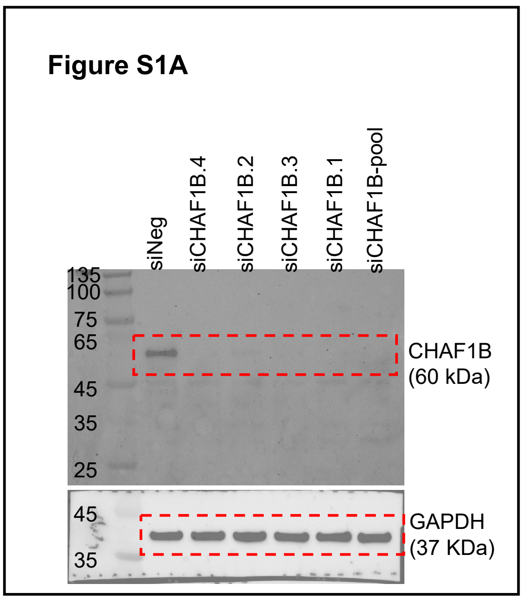

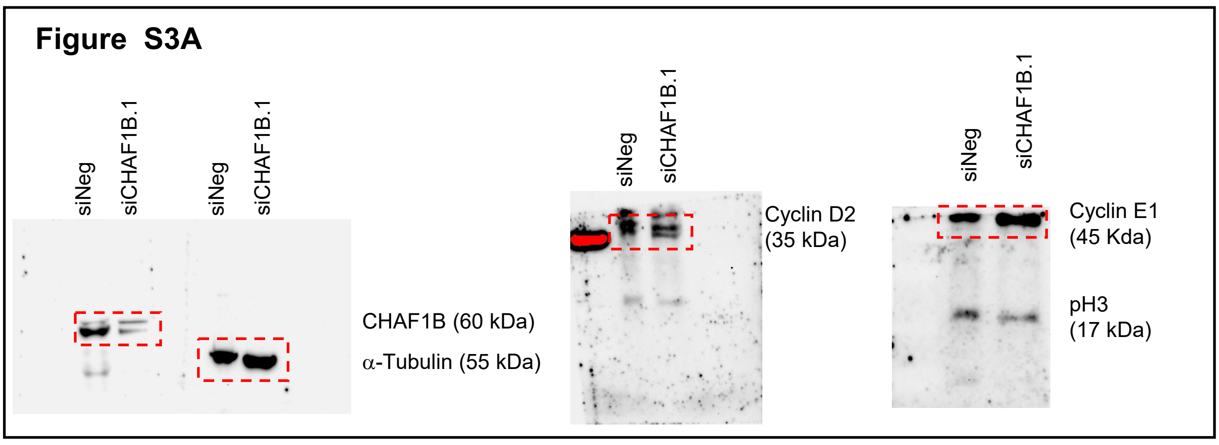

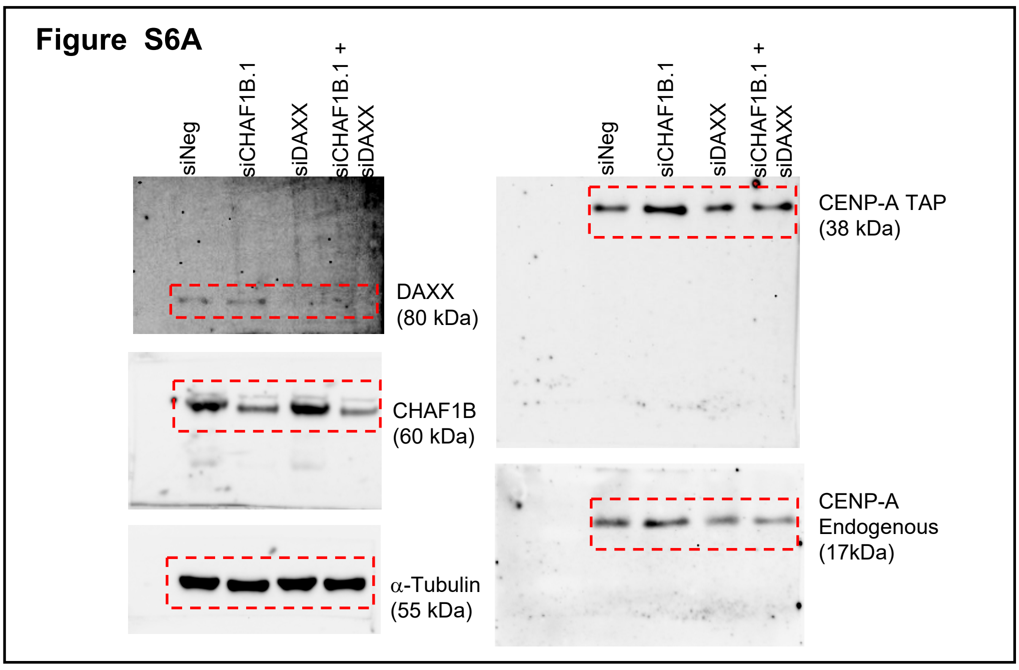

Fig. S7. Raw uncropped images of blots used in the paper. (A-C) Uncropped raw jpeg images of blots used in figures as indicated are shown. Dashed boxes represent the cropped images shown in the paper, and dashed black boxes represent the positions of cuts made in the membrane to probe different membrane sections with different antibodies .

Journal of Cell Science • Supplementary information

Table S1. siRNA sequences and primers sequences used in the study.

Click here to download Table S1

Table S2. List of genes and median score for each gene obtained from high-throughput siRNA screen.

Click here to download Table S2

Journal of Cell Science • Supplementary information
